# Zirconia Crowns for Primary Teeth: A Systematic Review and Meta-Analyses

**DOI:** 10.3390/ijerph19052838

**Published:** 2022-02-28

**Authors:** Sara Douf Alzanbaqi, Rakan Mishaal Alogaiel, Mohammed Ali Alasmari, Ahmed Mohammed Al Essa, Layla Nizar Khogeer, Basim Salem Alanazi, Eyad Sami Hawsah, Ahmed Mohammed Shaikh, Maria Salem Ibrahim

**Affiliations:** 1 Saudi Board of Pediatric Dentistry, College of Dentistry, King Abdulaziz University, Jeddah 21589, Saudi Arabia; saraalzanbaqi@gmail.com; 2 College of Dentistry, Imam Abdulrahman Bin Faisal University, Dammam 34212, Saudi Arabia; RAlogaiel@hotmail.com (R.M.A.); mohammadalirashed111@gmail.com (M.A.A.); Ahmed.m.alessa@hotmail.com (A.M.A.E.); basssiim.s@gmail.com (B.S.A.); ehawsah@gmail.com (E.S.H.); a.shaikh67856@gmail.com (A.M.S.); 3 Department of Pediatric Dentistry, College of Dentistry, King Abdulaziz University, Jeddah 21589, Saudi Arabia; laylakhogeer@yahoo.com; 4 Department of Preventive Dental Sciences, College of Dentistry, Imam Abdulrahman Bin Faisal University, Dammam 34212, Saudi Arabia

**Keywords:** zirconia crowns, pediatric dentistry, caries

## Abstract

Objective: The aim of this systematic review was to summarize the literature regarding the clinical performance of zirconia crowns for primary teeth. Materials and Methods: Four electronic databases, Ovid, PubMed, Scopus, and Web of Science were searched. Clinical, observational, and laboratory studies were included. Studies that assessed the performance of zirconia crowns for primary teeth using outcomes such as gingival and periodontal health, parental satisfaction, color stability, crown retention, contour, fracture resistance, marginal integrity, surface roughness, and recurrent caries were included. Risk of bias was assessed using different assessment tools depending on the type of the assessed study. Results: Out of the 2400 retrieved records, 73 full-text records were assessed for eligibility. Thirty-six studies were included for qualitative analysis. The included studies reported that zirconia crowns for primary teeth were associated with better gingival and periodontal health, good retention, high fracture resistance, color stability, high parental acceptance, good marginal adaptation, smooth cosmetic surface, and no recurrent caries. Conclusion: Zirconia crowns are promising alternative to other restorative materials and crowns in the field of pediatric dentistry. They showed higher properties and performance in different clinical aspects and great parental satisfaction.

## 1. Introduction

Dental caries is considered the most common infectious disease globally [1,2,3]. Internationally, 60–90% of children suffer from this disease [2,4]. When left untreated, caries could severely damage the tooth structure which will require restoration to one or more of the tooth surfaces. If it progresses further, the tooths pulp will be affected, and inflammation may result. At this stage, the tooth may require pulp therapy [5,6,7], and most probably the remaining tooth structure will need to be covered with a crown. This may be necessary to maintain the integrity of the treated tooth until the eruption of its permanent successor. Primary teeth play an important role in preserving space in the arch for the permanent teeth beside their important functions in speech and mastication [8]. For this reason, it is best to treat primary molars with extensive and large carious lesions, multiple affected surfaces or that have undergone pulp therapy with full coverage restorations or crowns. Full coverage is essential to provide long-term protection and durability and prevent recurrent decay [9].

The most widely recognized full coverage restoration method used in pediatric dentistry is the use of stainless steel crowns [10,11]. Stainless steel crowns are pre-formed metal crowns that have shown significant clinical success and are considered a favorable restoration method for multiple surfaces and larger carious lesions on primary molars [12,13,14]. Studies have evaluated the performance of stainless steel crowns in comparison to other restoration methods and found that stainless steel crowns showed a higher lifespan and durability [15,16,17,18]. The stainless steel crowns have reasonable costs and are less technique sensitive during placement [11,19].

Despite the favorable qualities mentioned above, stainless steel crowns have some drawbacks, including their poor esthetic appearance. This led their rejection by most parents as they are becoming more engaged in the treatment planning for their children and more considerate of their esthetic appearance [20,21,22,23]. In addition, tooth-colored restorations are preferred among children while silver-colored amalgam restorations are the least preferred [24,25].

Zirconia crowns were introduced in 2008 as an alternative restorative treatment. Zirconia has an extensive history of being an excellent biocompatible material [26]. One of the main advantages of zirconia crowns are their esthetically excellent appearance alongside their durability [27,28,29]. In addition, zirconia crowns have shown less plaque accumulation in comparison to other materials due to their highly polished surface [30,31]. However, there are some clinical limitations and disadvantages for zirconia crowns as they require aggressive tooth reduction and are expensive [27,32].

Zirconia as a material demonstrated excellent mechanical properties. Its flexural strength could reach up to 1200 MPa, and its toughness may reach up to 10 MPa [33,34]. When compared to porcelain-fused-to-metal crowns, zirconia crowns reported a higher strength which could reach to three times higher [33,34].

Zirconia crowns are relatively a new topic in pediatric dentistry. In this review, we aimed to review the literature systematically and explore the performance of zirconia crowns for primary teeth in clinical or laboratory settings. Different outcomes measures were considered for a comprehensive review.

## 2. Materials and Methods

### 2.1. Research Question

The review protocol was preset but not published. The Preferred Reporting Items for Systematic Reviews and Meta-Analyses (PRISMA) guidelines for systematic reviews and meta-analysis were followed. The PICO question of this systematic review was:

- Population: Primary teeth OR pediatric patients OR extracted teeth.
- Intervention: Pediatric zirconia crowns.
- Comparator: Other restorative materials OR crowns.
- Outcomes: Periodontal health, parental satisfaction, color stability, crown retention, contour, fracture resistance, marginal integrity, surface roughness, and recurrent caries.

### 2.2. Search Strategies

Four search strategies were built and applied for the following databases: PubMed, Web of Science, Scopus, and Ovid (Table 1). The last search was run on 5 January 2022. No date or language restriction was applied during the database searches.

### 2.3. Eligibility Criteria

In this systematic review, we included any relevant articles focused on prefabricated/ready-made zirconia crowns as permanent coverage crowns for primary teeth as interventions, with any other crown types or restorations as a comparison or no comparison. Clinical, observational, and laboratory in vitro studies were included with no restrictions used for language or the type of study. Clinical studies with special health care patients and studies on pediatric patients with permanent teeth only were excluded.

### 2.4. Studies Screening and Selection

The citations were then uploaded to the Covidence online platform for title and abstract screening. Two reviewers screened the titles and abstracts independently, and any conflict was resolved by a senior reviewer. The included citations were then screened as full texts.

### 2.5. Data Extraction

The data was extracted from the included studies by four reviewers. The extracted data included qualitative and quantitative data. The extracted data included publication date, sample size, size of each group, sex distribution, age, interventions, outcome parameters, and outcome findings.

### 2.6. Quality Assessment

The risk of bias of the included studies was assessed by two independent reviewers. The assessment tools were adapted the Cochrane assessment tools for the included clinical and observational studies and from previously published scoping and systematic reviews for the laboratory studies [35,36]. Clinical studies with one to two “Yes” only were considered to have a low risk of bias. Studies scoring three to four “Yes” or five to six “Yes” were considered to have a medium risk of bias or a high risk of bias, respectively. Observational studies with one to three “Yes” were considered to have a low risk of bias. Studies scoring four to five “Yes” or six to seven “Yes” were considered to have medium risk of bias or high risk of bias, respectively. Laboratory studies with one to three “Yes” were considered to have low risk of bias. Studies scoring four to six “Yes” or seven to eight “Yes” were considered to have moderate risk of bias or high risk of bias.

### 2.7. Data Synthesis

A qualitative summary of the included studies’ characteristics and findings was reported. We performed a quantitative meta-analysis using a fixed-effect model or a random-effect model if an I_2_ statistic at or below 50% was found with no significant methodological heterogeneity or an I_2_ statistic was found to be above 50% with no significant methodological heterogeneities, respectively. However, if significant statistical or methodological heterogeneity was found, a meta-analysis was not conducted.

## 3. Results

**Figure 1:**
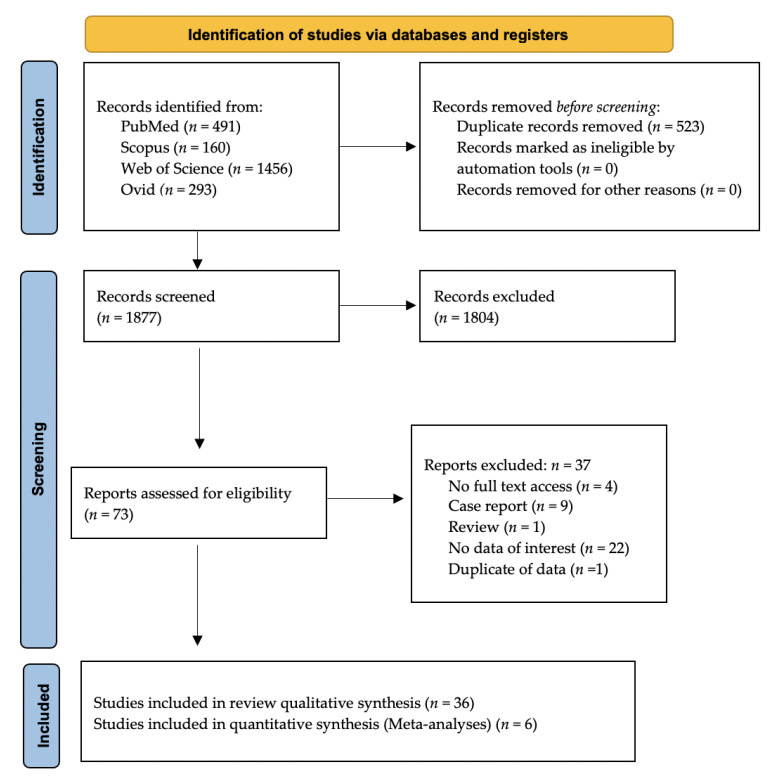
PRISMA 2020 flow diagram of the search results from the databases.

From the initial database searches, 2400 records were retrieved. Duplicates were removed, and 1877 records left for title and abstract screening. After title and abstract screening, full texts of 73 records were assessed for eligibility (Figure 1). Thirty-six studies were included in the final qualitative assessment, and six studies were included in the quantitative assessment.

### 3.1. Characteristics of the Included Studies

The characteristics of the included studies are presented in Table 2. The table included the type of study, sample size, outcome measures, interventions, and comparators. There was variation in the types of zirconia crowns used and evaluated in the included studies.

### 3.2. Quality Assessment of the Included Studies

**Figure 2:**
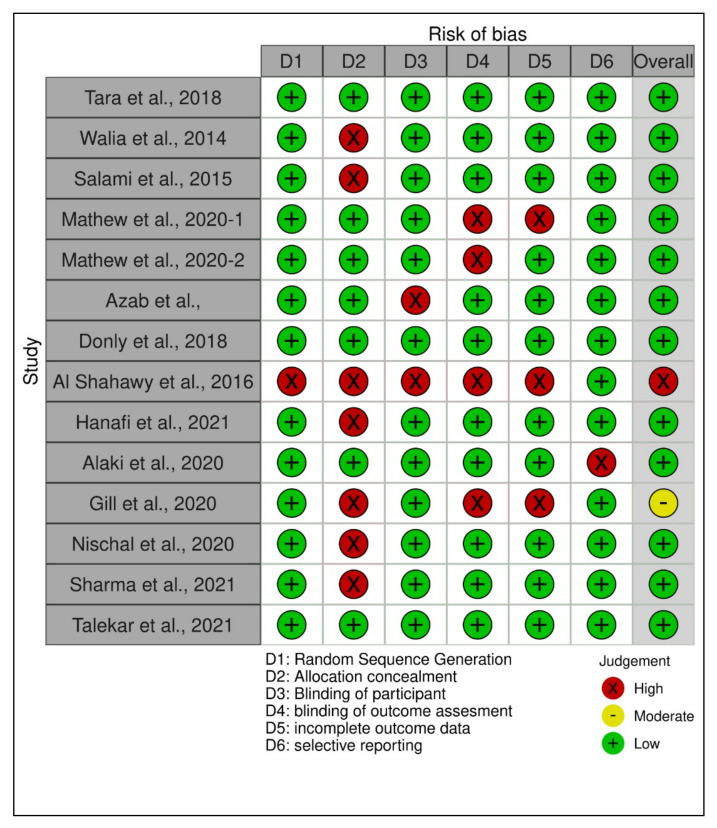
Individual study’s risk of bias appraisal for the included clinical studies.

**Figure 3:**
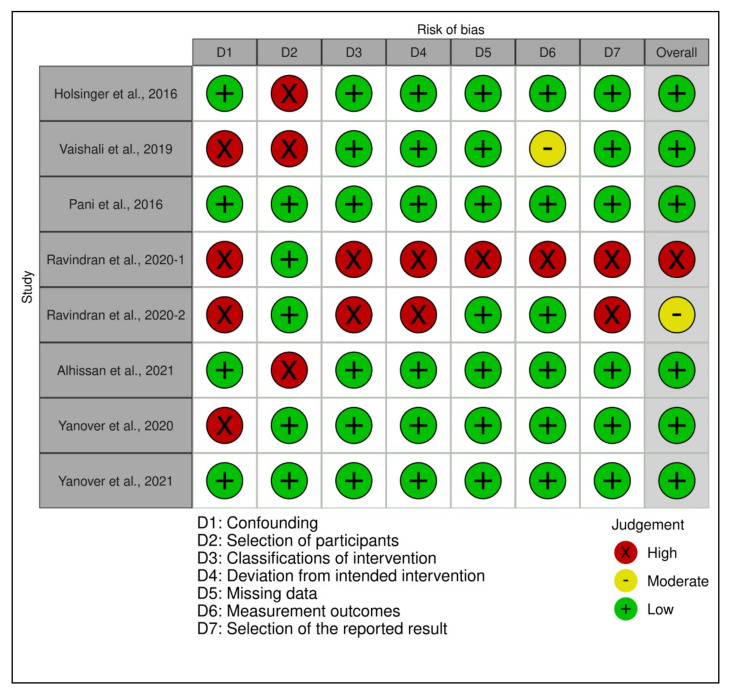
Individual study’s risk of bias appraisal for the included observational studies.

**Figure 4:**
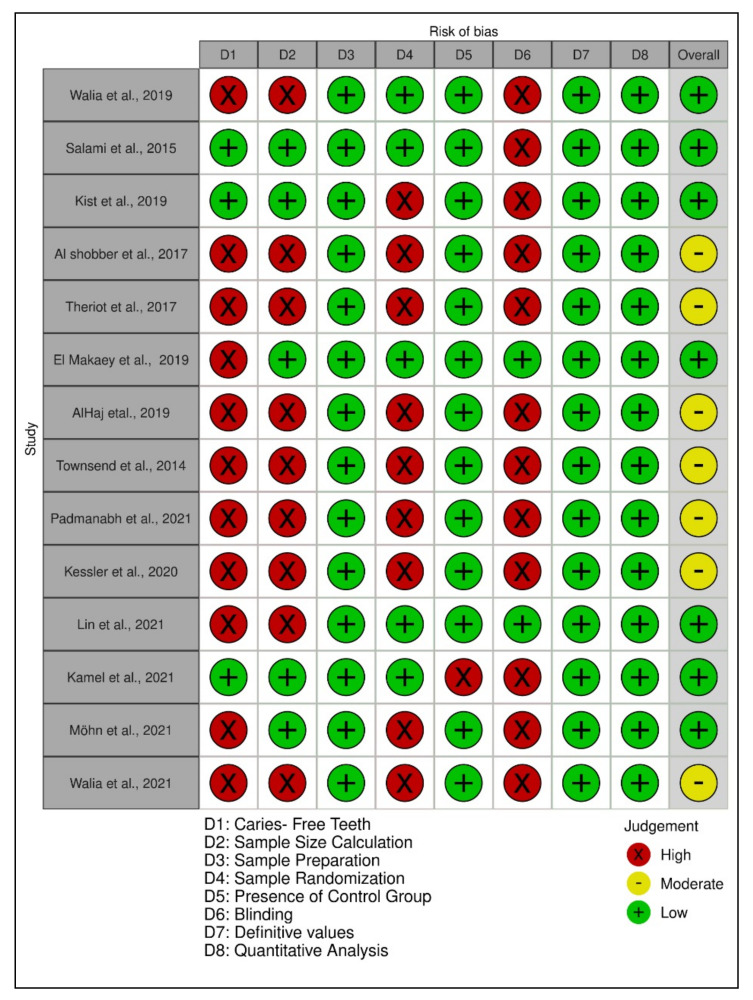
Individual study’s risk of bias appraisal for the included laboratory studies.

**Figure 5:**
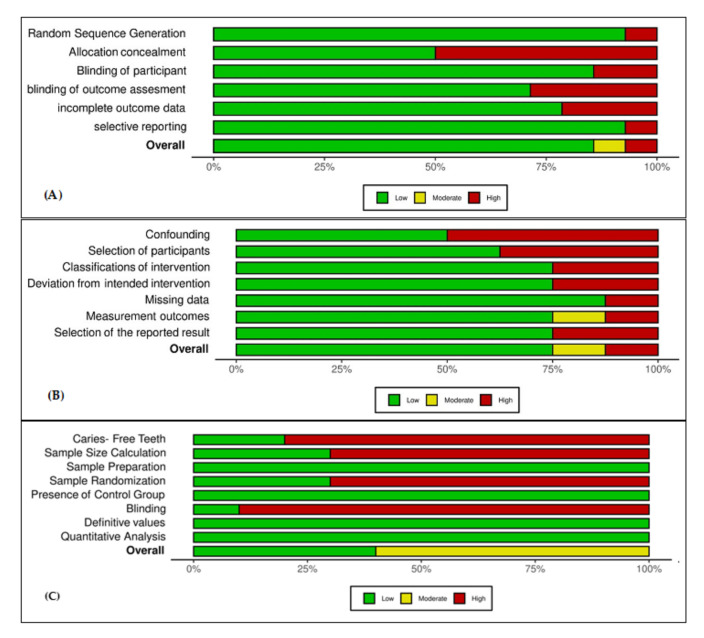
Risk of bias appraisal for each parameter. (**A**) Clinical studies. (**B**) Observational studies. (**C**) Laboratory studies.

The quality assessments of the individual included studies are shown in Figure 2, Figure 3 and Figure 4. The overall quality assessments of the existing evidence based on the type of included studies are presented in Figure 5.

### 3.3. Gingival and Periodontal Health

Thirteen studies assessed gingival and periodontal health when placing different types of zirconia crowns. The assessment time varied between 1 week and 36 months after crown placement. Two studies [50,53] showed no significant differences in gingival index and periodontal index while four studies [27,30,42,62] showed significant differences in both indices between different types of crowns or restorations in comparison to zirconia crowns. A summary of the findings of each study regarding this outcome is presented in Supplemental Table S1.

### 3.4. Parental Satisfaction

Eight studies evaluated the level of parental satisfaction of zirconia crowns. It was shown that zirconia crowns had a higher satisfaction rate than different control groups in all studies [21,37,40,41,43,59,64,66]. Supplemental Table S2 shows the details of the findings for this outcome.

### 3.5. Color Stability

Nine studies investigated the color stability and stain resistance when using zirconia crowns. The evaluation time was between 1 and 36 months after crown placement. All studies reported high color stability and stain resistance of zirconia crowns [30,37,43,51,54,59,62,63,64]. Two randomized clinical trials showed no significant differences between zirconia crowns and control groups [51,62]. Supplemental Table S3 illustrates the detailed findings about the color stability of zirconia crowns.

### 3.6. Crown Retention

Thirteen studies assessed the retention of zirconia crowns. The assessment time varied between 1 week and 36 months after crown placement. One randomized clinical trial showed that zirconia crowns had a statistically significant higher retention rate when using packable glass ionomer [50]. Two randomized clinical trials showed a statistically significantly higher retention rate of zirconia crowns when compared to the control groups [27,62]. An additional description of the findings for this outcome is shown in Supplemental Table S4.

### 3.7. Fracture Resistance

Eleven studies evaluated the fracture resistance of zirconia crowns. The evaluation time was between 1 week and 36 months. One randomized clinical trial showed high fracture resistance of zirconia crowns [30], and two laboratory studies proved that zirconia crowns required high fracture loads to break in comparison to the control groups [45,47]. Supplemental Table S5 gives more information about the findings.

### 3.8. Marginal Integrity

Eight studies assessed the marginal integrity of zirconia crowns. The assessment time ranged from 3 to 33.8 months. One laboratory study showed that zirconia crowns cemented with resin cement had a statistically significant lower internal gap width than the control group [48]. and four studies proved that zirconia crowns have high marginal adaptation and were clinically ideal [37,51,60,63,69]. A summary of the results from different studies is provided in Supplemental Table S6.

### 3.9. Surface Roughness

Four studies investigated the presence of surface roughness among zirconia crowns. One randomized clinical trial showed that all zirconia crowns exhibited a smooth surface except two crowns that showed slight roughness but were clinically acceptable. However, the difference was not statistically significant when compared to the control group [51]. A summary and details of the results are provided in Supplemental Table S7.

### 3.10. Recurrent Caries

Four studies evaluated the presence of recurrent caries with different types of zirconia crowns. The follow-up time ranged from 3 to 24 months. It was shown that zirconia crowns did not cause recurrent caries in all included studies. One study showed a statistically significant difference between zirconia crowns and the control groups [60]. Supplemental Table S8 shows the details of the results.

### 3.11. Crown Contour

Two retrospective studies assessed the crown contour of zirconia crowns, and the majority were natural looking and cosmetic [37,63]. Supplemental Table S9 summarizes the findings.

### 3.12. Meta-Analyses

**Figure 6:**
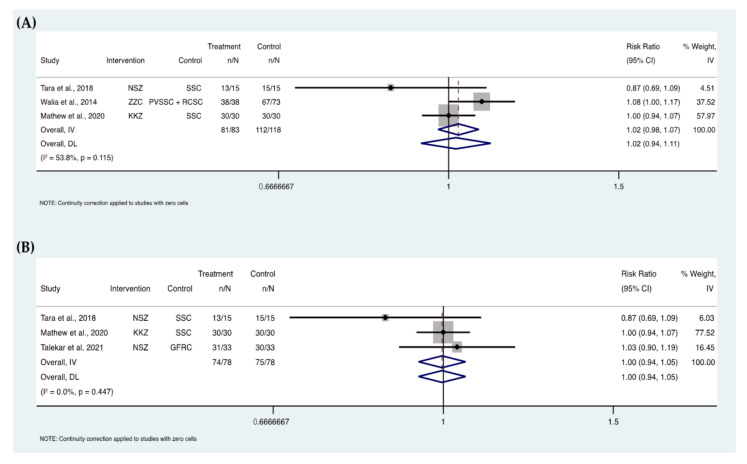
Forest plots of the retention of zirconia crowns at 6 (**A**) and 12 (**B**) months (NSZ, NuSmile zirconia crown; ZZC, Zirkiz zirconia crown; KKZ, Kinder Krown zirconia crown; SSC, stainless steel crown; RCSC, resin composite strip crown; PVSSC, pre-veneered stainless steel crown; GFRC; glass fiber-reinforced composite crown—Figaro Crowns).

**Figure 7:**
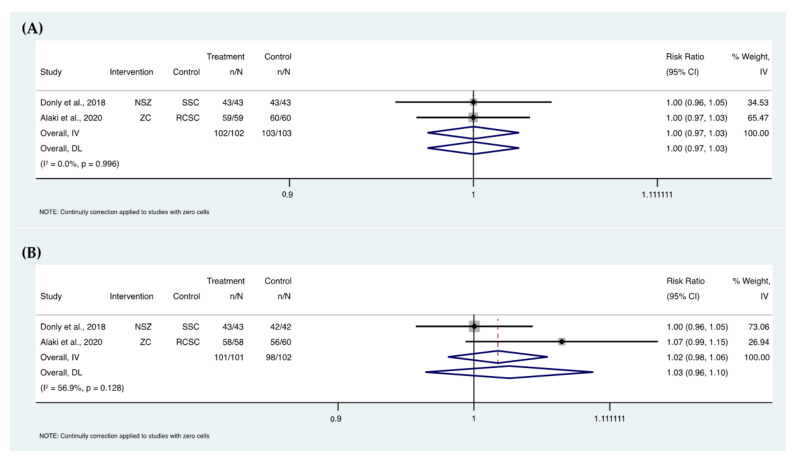
Forest plots of recurrent caries of zirconia crowns at 6 (**A**) and 12 (**B**) months (NSZ, NuSmile zirconia crown; SSC, stainless steel crown; ZC, zirconia crown; RCSC, resin composite strip crown).

Four meta-analyses were performed (Figure 6 and Figure 7). Two analyses included three studies [27,30,43,64] and two analyses included two studies [30,43,51,57]. The quantitative grouping of these studies showed no differences between zirconia crowns and their control groups in the two compared outcomes: crown retention and recurrent caries. This was based on the clinical results for the retention at 6 (relative risk (RR) = 1.02, 95% CI, 0.94–1.11, *p* = 0.115; I_2_ = 53.8%) and 12 (RR = 1.00, 95% CI, 0.94–1.05, *p* = 0.447; I_2_ = 0%) months, and for the recurrent caries at 6 (RR = 1.00, 95% CI, 0.97–1.03, *p* = 0.996; I_2_ = 0%) and 12 (RR = 1.03, 95% CI, 0.96–1.10, *p* = 0.128; I_2_ = 56.9%) months.

## 4. Discussion

Zirconia crowns for primary teeth are in high demand from parents who seek more esthetically pleasant dental restorations for their children. Research has been undertaken to compare the properties of zirconia crowns for primary teeth with other similar restorations such as stainless steel crowns. This systematic review aimed to summarize the performance of zirconia crowns for primary teeth by reporting the findings in the literature of 3575 teeth that were included regarding their different clinical aspects and parental satisfaction. These clinical aspects include gingival and periodontal health, color stability, retention, fracture resistance, marginal integrity, restoration failure, surface roughness, recurrent caries, and crown contour.

Zirconia crowns are indicated as the same as any other available type of crown in pediatric dentistry. However, there are some potential drawbacks of zirconia crowns such as the difficulty of adjustments to provide mechanical retention in contrast to stainless steel crown, the limitation of the shades available in the clinics, and the prolonged procedure time. The zirconia crowns require more tooth structure reduction to accomplish better adaptation. Pulpal exposure and postoperative complications also have been noted during the preparation for zirconia crowns [32]. Even with the variety of companies and esthetic demands, zirconia crowns are considered to be expensive when compared to other treatment alternatives [32,33].

One of the important parameters to assess in a crown is its effect on gingival and periodontal health. An ideal material for a crown would have no plaque accumulation on the surface. Different materials used for crowns may have different properties leading to different plaque accumulation amounts. Other factors such as types of cements also may affect periodontal health. In this review, we found that most of the included studies found that zirconia crowns had significantly lower levels of plaque accumulation, especially when compared to resin-coated crowns [57]. This could be due to the surface properties of zirconia including its superior hardness. This makes them resistant to scratches and they may have a shiny, smooth polished surface. Another reason could be the low surface energy of zirconia crowns which may lead to low plaque and bacterial adhesion. Although, if the plaque accumulated on the surfaces, it was reported to be thinner than the plaque on stainless steel crowns [42,70]. This is due to the smoother surfaces and margins of zirconia crowns unlike stainless steel crowns or strip crowns which require a customization and recontouring before cementation. The recontouring or adjustments may create irregularities on surfaces and margins, favoring the accumulation of plaque and affecting periodontal health [30]. Therefore, zirconia is being used for a variety of applications such as implants [71]

In this review, nearly all included studies showed greater parental acceptance of zirconia crowns compared to other treatment modalities, even when other esthetic restorations such as pre-veneered stainless steel crowns were offered or used. Zirconia crowns scored the highest satisfaction rates for the parents and their children [13,21,37,40]. This shows that although the process of preparing crowns is prolonged, the esthetic component of a restoration is important for parents [37,72]. This is an important point for the clinician to consider when offering treatment plan options. This is important especially for anterior teeth where esthetics is of high importance [72].

The review findings also showed that zirconia crowns have a high degree of color stability. This factor can be considered when offering zirconia as an esthetic solution to parents when compared to other crowns such as the resin-coated stainless steel crowns. Zirconia crowns exhibit a highly polished surface that prevents staining and color deterioration [59]. With sterilization techniques, zirconia crowns showed no color changes along with crazing or fractures which was the lowest of the tested groups (stainless steel crowns and pre-veneered stainless steel crowns) [54].

The color change of the latter can negate the original purpose of the resin as an esthetic solution as resin is prone to staining over time when exposed to agents such as coffee or dark soft drinks [73,74].

Although some studies have shown that the stainless steel crowns have a higher retention rate than the zirconia crown, the retention of the zirconia crown is acceptable. This is because the clinician is unable to crimp and counter the crown clinically to adapt it to the tooth and must rely upon the prefabricated form of the crown. This is also considering the relatively short period of time that the restoration will be in the patient mouth as the deciduous tooth will be exfoliated in a couple of years. Regardless, stainless steel crowns facilitate its retention through crimping and contouring while zirconia crowns require greater tooth reduction to create more surface area for cement anchorage [13,32,39]. Furthermore, superior retention was found in zirconia crowns when compared to other esthetic crowns such as Luxa crown and strip crowns [60]. Different zirconia crowns from different manufacturers have different methods of retention. Zirconia crowns by NuSmile are different from others by having no grooves on their inner surface. On the other hand, zirconia crowns such as the ones by Kinder Krown have grooves in their inner occlusal and axial surfaces to improve retention [75]. These grooves are wider in EZCrowns [75,76]. In this systematic review, zirconia crowns by Kinder Krown, NuSmile, and EZCrowns showed acceptable levels of retention when compared to other restorations or crowns.

The included studies in this review showed high fracture resistance of zirconia crowns for primary teeth. This may make them a good alternative to resin restorations in patients with grinding habits. Although some studies suggest that ceramic compounds can produce a degree of wear on the opposing teeth [77], a review of the literature indicates that zirconia crowns do not cause this phenomenon [78]. One area of concern for zirconia crowns for primary teeth is the fact they are prefabricated and are not custom-made for the patients’ teeth. Therefore, marginal adaptation and integrity may be compromised. This review showed that using resin cement may be recommended due to the cement acting as a barrier in less ideally adapted margins [48]. Other in vitro studies have corroborated this fact [79]. Even with zirconia crown in a prefabricated state, four studies proved that zirconia crowns have high marginal adaptation and were clinically acceptable [37,51,60,63]. A 12 months study period revealed that zirconia crowns and stainless steel crowns had better marginal adaptation along with facing integrity than composite strip crowns [59]. The surface of zirconia crowns may show some roughness according to our review although they are clinically acceptable. Our review also showed that zirconia crowns have a high deal of success with a low rate of recurrent caries.

In this review, meta-analyses were conducted on two parameters: the retention of zirconia crowns and the rate of recurrent caries. Both parameters were comparable to control treatments used in the included studies. However, more randomized clinical trials are recommended as only a few studies were included in these analyses as most of the clinical trials did not have control groups to compare the performance of zirconia crowns for primary teeth. The follow-up periods in the included studies in the meta-analyses were at 6 and 12 months. The total number of the included zirconia crowns assessed for retention were 83 crowns at 6-month follow-up and 78 crowns at the 12-month follow-up period with a total of 118 control crown/teeth at 6-month and 78 crowns/teeth at 12-month follow-up periods. For the recurrent caries assessment, intervention and control groups were almost similar in the number of included crowns/teeth which was around 100.

It is important to point out the variation between the included studies in terms of age groups of the included patients. It was observed that participants’ ages ranged from three to nine years old. Additionally, the variation in the study types ranging from split-mouth design to observational design should be considered as this may affect the interpretation of the findings.

This review was limited by several factors including the focus on only articles in English language and the lack of search in gray literature which may leave some evidence unavailable. However, this review included a larger number of studies than the previously published review [78]. Additionally, this review covered a wider range of outcome measures and clinical aspects regarding the performance of zirconia crowns for primary teeth.

## 5. Conclusions

In conclusion, zirconia crowns are a promising alternative to other restorative materials and crowns in the field of pediatric dentistry. They showed greater properties and performance in terms of different clinical aspects and great parental satisfaction. However, there is a need for more randomized clinical trials that assess the various clinical aspects of primary teeth zirconia crown performance in comparison to other types of crowns or restorations for primary teeth. Additionally, further clinical studies with longer follow-up periods are needed. When considering zirconia crowns as an alternative to other materials and crowns for primary teeth, length of procedure, expensive cost, and dentist skills should be considered, especially for primary teeth.

## Figures and Tables

**Table 1:** Search strategies.

Database: PubMed	Results
((child*)[tiab] OR (Primary)[tiab] OR (deciduous)[tiab] OR (tooth, deciduous)[MeSH] OR (Pediatric)[tiab] OR (Paediatric)[tiab])) AND ((zirconia)[tiab]))	491
**Database: Scopus**	**Results**
(TITLE-ABS-KEY ((child* OR deciduous OR pediatric*))) AND (TITLE-ABS-KEY (zirconia))	160
**Database: Web of Science**	**Results**
((child*) OR (Primary) OR (decisdous) OR (tooth, deciduous) OR (Pediatric) OR (Paediatric) AND (zirconia)	1456
**Database: Ovid**	**Results**
((child* or primary or deciduous or pediatric* or paediatric).af.) AND (zirconia.af.)	293

**Table 2:** Included studies’ characteristics.

Study	Type of Study	Sample Size per Group	Participant Characteristics	Outcome Measures	Intervention	Comparator	Cement Type
Taran et al., 2018 [30]	Clinical	15	Age (A) = 6–9 Y Number of patients (T) = 15 Female (F) = 9 Male (M) = 6	Crown retention Gingival marginal extension Stain resistance Fracture resistance Plaque index (PI) Gingival index (GI) Simplified oral Hygiene index	NuSmile zirconia crown (NSZ)	Intact contralateral teeth stainless steel crown (SSC)	SSC: Glass ionomer cement (GIC) NSZ: Resin modified glass ionomer cement (RMGIC)
Walia et al., 2014 [27]	Clinical	43	A = 3–5 Y T = 39 M = 21 F = 18	Crown retention Tooth wear GI	Zirkiz zirconia Crown (ZZC)	Resin Composite Strip Crown (RCSC) Pre-veneered stainless steel crown (PVSSC)	RCSC: (3M, Scotchbond-Universal-Adhesive-Refill-Vial-41258^®^) PVSSC: GIC-II ZZC: GIC-II
Holsinger et al., 2016 [37]	Observational	57	A = 2–6 Y T = 18 F = 6 M = 12	Crown retention GI Stain resistance Crown contour Marginal integrity Tooth wear Recurrent caries Parent acceptability	EZ Pedo crown (EZP)	-	EZP: GIC
Walia et al., 2019 [38]	Laboratory	10	-	Surface roughness	NSZ Spring EZ crown (SEC) Cheng crown zirconia (CCZ) Kinder Krown zirconia crown (KKZ)	-	-
Salami et al., 2015 [21]	Observational	43	A = 3–5 Y T = 39 F = 18 M = 21	Parental satisfaction	ZZC	RCSC PVSSC	-
Jing et al., 2019 [39]	Laboratory	15	-	Crown retention of zirconia Crown (ZC) for primary teeth with various Occluso-Cervical Hights (OCH) crown preparation	SEC	-	SEC: GIC
Vaishali et al., 2019 [40]	Observational	125	A = 6–8 Y	Parent acceptability	Questionnaire	-	-
Pani et al., 2016 [41]	Observational	107	A = 5–8 Y	Parent acceptability	Questionnaire	-	-
Mathew et al., 2020 [42]	Clinical	30	A = 6–9 Y T = 30 F = 18 M = 12	GI PI CFU/mL count of S. *mutans*	KKZ	SSC	-
Mathew et al., 2020 [43]	Clinical	30	A = 6–8 Y	Crown retention GI PI Stain resistance Gingival marginal extension Occlusion Proximal contact Parent acceptability	KKZ	SSC	All: GIC-I
Kist et al., 2019 [44]	Laboratory	85	-	Fracture resistance	SEC KKZ NSZ	Computer-aided manufacturing/computer-aided modeling zirconia crown (CAD/CAM) ZC PVSSC SSC	All: GIC
Al shobber et al., 2017 [45]	Laboratory	16	-	Fracture resistance	CCZ NSZ	PVSSC Cheng crown pre-veneered (CCP)	All: GIC
Theriot et al., 2017 [46]	Laboratory	20	-	Surface roughness Surface gloss	NSZ SEC KKZ	-	-
El Makawi et al., 2019 [47]	Laboratory	10	-	Fracture resistance	NSZ	Lithium disilicate endocrown (LDE)	All: resin composite (RC)
Alhaj et al., 2019 [48]	Laboratory	12	-	Marginal and internal gap	NSZ	SSC PVSSC	All: RC, GIC, or RMGIC
Townsend et al., 2014 [49]	Laboratory	20	-	Fracture resistance Crown thickness	EZP NSZ KKZ	PVSSC	All: GIC
Azab et al. [50]	Clinical	25	A = 4–7 Y T = 25 F = 11 M = 14	Crown retention Fracture resistance GI	NSZ	Different types of cements	NSZ: GIC-IX or bioactive cement
Donly et al., 2018 [51]	Clinical	50	A = 3–7 Y	GI Occlusion Surface roughness Stain resistance Tooth wear Color match Anatomic form Marginal integrity Marginal discoloration Proximal contact Recurrent caries	NSZ	SSC	NSZ: Bioceramic Cement SSC: RMGIC
El Shahawy et al., 2016 [52]	Clinical	86	A = 2–5 Y	Crown Retention	NSZ	-	NSZ: GIC-IX
Hanafi et al., 2021 [53]	Clinical	(CAD/CAM) ZC = 31 NSZ = 32	A = 5–9 Y T = 44 F = 16 M = 28	GI PI Bleeding on probing (BOP) Crown marginal extension	(CAD/CAM) ZC	NSZ	All: GIC
Padmanabh et al., 2021 [54]	Laboratory	20	-	Stain resistance Crazing Dimensional stability Fracture resistance	KKZ	SSC PVSSC	-
Ravindran et al., 2020 [55]	Observational	107	A = 2–7 Y T = 107 F = 42 M = 65	Prevalence	ZC	RCSC SSC	-
Ravindran et al., 2020 [56]	Observational	1496	A = 0–10 Y T = 1496 F = 628 M = 868	Prevalence	NSZ	SSC	All: Type I GIC
Alaki et al., 2020 [57]	Clinical	60	A = 4–6 Y T = 32 F = 20 M = 12	GI PI Recurrent caries Restoration failure Proximal contact Marginal integrity Occlusion Tooth wear	ZC	RCSC	ZC: RC
Alhissan et al., 2021 [58]	Observational	70	A = 3–5 Y T = 20 F = 11 M = 9	Restoration failure	NSZ	With/without pulp therapy	-
Gill et al., 2020 [59]	Clinical	135	A = 2–4 Y T = 47	Crown fit Proximal contact Color match Crown retention Facing integrity Marginal integrity GI Recurrent caries Parent satisfaction	NSZ	RCSC PVSSC	RCSC: (Scotchbond Universal, 3M ESPE, St. Paul, MN, USA) PVSSC: GIC NSZ: RMGIC
Nischal et al., 2020 [60]	Clinical	45	T = 45	Surface roughness Anatomical form Marginal integrity Marginal discoloration Recurrent caries	ZC	RCSC Luxa crown	RCSC: bonding agent ZC: RMGIC Luxa crown: RMGIC
Kessler et al., 2020 [61]	Laboratory	-	-	Crown wear Fracture	NSZ	Composite crown SSC	All: RMGIC and two self-adhesive cements (SACs; RelyX Unicem Automix 2, 3M; BioCem, NuSmile)
Sharma et al., 2021 [62]	Clinical	20	A = 3–5 Y T = 24	GI PI Tooth wear Color Restoration failure	ZC	RCSC	RCSC: Light cure bonding adhesive (3M, Scotchbond-Universal Adhesive-Refill-Vial-41258^®^) ZC: Type II GIC
Yanover et al., 2020 [63]	Observational	131	A = 2–5 Y T = 36 F = 5 M = 31	Marginal integrity GI Restoration failure	SEC NSZ CCZ	-	-
Talekar et al. 2021 [64]	Clinical	33	A = 4–9 Y T = 30	Color match Stain resistance GI Crown retention PI Occlusal wear Parent satisfaction	NSZ	Glass fiber-reinforced composite crown—Figaro Crowns (GFRC)	NSZ: RMGIC GFRC crowns: Type I GIC
Lin et al., 2021 [65]	Laboratory	15	-	Fracture resistance	EZP Polycarbonate crowns—PedoNatural RCSC	-	EZP: Type I GIC and self-adhesive resin cement (RelyX Unicem, 3M ESPE Polycarbonate crowns: polymer-reinforced zinc oxide-eugenol cement (IRM Dentsply).
Yanover et al., 2021 [66]	Observational	131	A = 2–5 Y T = 37 F = 10 M = 27	Parent satisfaction	EZP NSZ CCZ	-	-
Walia et al., 2021 [67]	Laboratory	24	-	Crown retention	NSZ SEC KKZ CCZ	-	FujiCEM^®^ 2 (GC America, Alsip, IL, USA) KetacTM Cem Maxicap (3M ESPE, St. Paul, MN, USA) BioCem (NuSmile, Houston, TX, USA)
Sabbah et al., 2020 [68]	Laboratory	6	-	Fracture resistance	NSZ	Nano-Ceramic Composite Endocrowns	NSZ: GIC NCCE: self-adhesive universal dual cured resin cement
Mohn et al., 2021 [69]	Laboratory	144	-	Marginal integrity Tooth wear Crown fracture	RCSC (CAD/CAM) ZC	SSC ZC	All: GIC RMGIC dual-cure self-adhesive resin cement (SAC) RC

A, age; T, total number of patients; M, male; F, female; GI, gingival index; PI, plaque index; NSZ, NuSmile zirconia crown; SSC, stainless steel crown; GIC, glass ionomer cement; RMGIC, resin modified glass ionomer cement; ZZC, Zirkiz zirconia crown; RCSC, resin composite strip crown; PVSSC, pre-veneered stainless steel crown; EZP, EZ Pedo crown; SEC, Spring EZ crown; CCZ, Cheng crown zirconia; KKZ, Kinder Krown zirconia crown; ZC, zirconia crown; OCH, Occluso-Cervical Heights; CAD/CAM ZC, computer-aided manufacturing/computer-aided modeling zirconia crown; CCP, Cheng crown pre-veneered; LDE, lithium disilicate endocrown; RC, resin composite; BOP, bleeding on probing; GFRC, glass fiber-reinforced composite crown—Figaro crowns.

## Data Availability

No new data were created or analyzed in this study. Data sharing is not applicable to this article.

## Supplementary Materials

The following are available online at https://www.mdpi.com/article/10.3390/ijerph19052838/s1, Table S1. Summary of the included studies’ findings about zirconia crowns for primary teeth and gingival and periodontal health; Table S2. Summary of the included studies’ findings about parental satisfaction with zirconia crowns for primary teeth; Table S3. Summary of the included studies’ findings about the color stability of zirconia crowns for primary teeth; Table S4. Summary of the included studies’ findings about the retention of zirconia crowns for primary teeth; Table S5. Summary of the included studies’ findings about the fracture resistance of zirconia crowns for primary teeth; Table S6. Summary of the included studies’ findings about the marginal integrity of zirconia crowns for primary teeth; Table S7. Summary of the included studies’ findings about the surface roughness of zirconia crowns for primary teeth; Table S8. Summary of the included studies’ findings about recurrent caries with zirconia crowns for primary teeth; Table S9. Summary of the included studies’ findings about the crown contour of zirconia crowns for primary teeth.
