# Supplementary material for: Zirconia Crowns for Primary Teeth: A Systematic Review and Meta-Analyses
Source: Int J Environ Res Public Health. 2022 Feb 28;19(5):2838. doi: 10.3390/ijerph19052838 (PMC8910015; doi:10.3390/ijerph19052838)
Supplement: Supplementary file 1 [file ijerph-19-02838-s001.zip › ijerph-1532405-supplementary.pdf]

International Journal of
Environmental Research and Public Health

MDPI

Supplemental Table S1. Summary of the included studies' findings about zirconia crowns for primary teeth and gingival and periodontal health.

<table>
<tr><th>Study</th><th>Assessment Method</th><th>Time of Evaluation</th><th>Results</th><th>Summary of Results</th></tr>
<tr><td>Tara et al., 2018 [30]</td><td>PI:
0= None
1= Film at margins
2= Moderate
3= Abundance
GI:
0= None
1= Mild
2= Moderate
3= Severe</td><td>At 1, 3, 6, 12 m</td><td>
<table>
<tr><th colspan="7">Median GI</th></tr>
<tr><th>Crown Type</th><th>Baseline</th><th>1 m</th><th>3 m</th><th>6 m</th><th>12 m</th><th>p-value</th></tr>
<tr><td>ZC</td><td>0.25</td><td>0.25</td><td>0.25</td><td>0.25</td><td>0.25</td><td>0.076</td></tr>
<tr><td>C-ZC</td><td>0.5</td><td>1</td><td>0.25</td><td>0.75</td><td>0.5</td><td>0.0003</td></tr>
<tr><td>SSC</td><td>0.5</td><td>1</td><td>0.75</td><td>1</td><td>1</td><td>0.034</td></tr>
<tr><td>C-SSC</td><td>0</td><td>0.25</td><td>0.25</td><td>0.5</td><td>0.5</td><td>0.004</td></tr>
<tr><th colspan="7">Median PI</th></tr>
<tr><th>Crown Type</th><th>Baseline</th><th>1 m</th><th>3 m</th><th>6 m</th><th>12 m</th><th>p-value</th></tr>
<tr><td>ZC</td><td>0.25</td><td>0</td><td>0</td><td>0</td><td>0</td><td>0.18</td></tr>
<tr><td>C-ZC</td><td>0.5</td><td>0.75</td><td>0.5</td><td>0.75</td><td>0.75</td><td>0.124</td></tr>
<tr><td>SSC</td><td>0.5</td><td>0.5</td><td>1</td><td>1</td><td>1</td><td>0.257</td></tr>
<tr><td>C-SSC</td><td>0.25</td><td>0.75</td><td>0.5</td><td>0.5</td><td>0.5</td><td>0.55</td></tr>
</table>
</td><td>There were significantly different GI scores among the C-ZC, SSC, and C-SSC groups at all evaluation time-points (C-ZC P=0.0003, SSC P=0.034, C-SSC P=0.004) respectively, no significant difference was found in ZC group (P=0.076). There was significant difference between the ZC and their controls in PI and GI scores at all evaluations times after baseline (P<0.05). Whereas in SSCs, there were no significant difference in PI scores at all evaluation times after baseline. However, the GI scores were all significantly different except at 1-month follow up (P<0.05).</td></tr>
<tr><td>Walia et al., 2014 [27]</td><td>GI:
0= None
1= Mild
2= Moderate
3= Severe</td><td>At 6 m</td><td>
<table>
<tr><th colspan="5">Mean GI</th></tr>
<tr><th></th><th></th><th></th><th>Baseline</th><th>6 m</th></tr>
<tr><th>Crown Type</th><th>n-Baseline</th><th>n-6m</th><th>Mean (SD)</th><th>Mean (SD)</th></tr>
<tr><td>RCSC</td><td>43</td><td>36</td><td>1.65 (0.50)</td><td>1.97 (0.44)</td></tr>
<tr><td>PVSSC</td><td>43</td><td>37</td><td>1.71 (0.50)</td><td>1.95 (0.61)</td></tr>
<tr><td>ZZC</td><td>43</td><td>38</td><td>1.67 (0.56)</td><td>1.35 (0.60)</td></tr>
</table>
</td><td>The Results showed that at the 6-month follow up, the mean GI was increased in group RCSC and PVSSC, while it was significantly reduced (p=0.01) in ZZC. When comparing the differences mean GI between all groups, there was statistically significant differences between RCSC and ZZC and PVSSC and ZZC (p=0.00) only.</td></tr>
</table>

<table>
<tr><td></td><td></td><td></td><td></td><td></td></tr>
<tr>
<td>Holsinger et al., 2016 [44]</td>
<td>GI:
0= None
1= Mild
2= Moderate
3= Marked</td>
<td>(Retrospective study) 2010 – 2014</td>
<td>Total number of teeth in each group (N) = 44
<table>
<tr><th>Score</th><th>0</th><th>1</th><th>2</th><th>3</th></tr>
<tr><td>n (%)</td><td>28 (64)</td><td>14 (32)</td><td>2 (4)</td><td>0 (0)</td></tr>
</table></td>
<td>The results showed that (36%) of EZP showed a mild to moderate gingival inflammation.</td>
</tr>
<tr>
<td>Mathew et al., 2020 [48]</td>
<td>GI:
0= None
1= Mild
2= Moderate
3= Severe
PI:
0= None
1= Film at margins
2= Moderate
3= Large amount</td>
<td>At 3, 6, 9, and 12 m</td>
<td>Total number of teeth in each group (N) = 30
Mean (SD)
<table>
<tr><th>Crown Type</th><th>3 m</th><th>6 m</th><th>9 m</th><th>12 m</th></tr>
<tr><td>PI KKZ</td><td>0.80 (0.1)</td><td>0.95 (0.1)</td><td>0.99 (0.1)</td><td>1.01 (0.1)</td></tr>
<tr><td>GI KKZ</td><td>0.80 (0.1)</td><td>1.12 (0.2)</td><td>1.47 (0.1)</td><td>1.76 (0.1)</td></tr>
<tr><td>PI SSC</td><td>1.48 (0.2)</td><td>1.75 (0.1)</td><td>1.92 (0.1)</td><td>2.41 (0.1)</td></tr>
<tr><td>GI SSC</td><td>1.38 (0.1)</td><td>1.63 (0.2)</td><td>1.89 (0.1)</td><td>2.11 (0.3)</td></tr>
</table></td>
<td>The results showed that KKZ reported a significantly lower GI and PI scores at all time-points compared to SSC (P<0.05).</td>
</tr>
<tr>
<td>Mathew et al., 2020 [49]</td>
<td>GI:
0= Healthy
1= Mild, involving some papilla
2= Moderate, involving entire papilla
3= Severe
PI
0= None
1= Film at margin
2= Moderate
3= Large amount</td>
<td>At 6, 12, 18, 24, and 36 m</td>
<td>Total number of teeth in each group (N) = 30
<table>
<tr><th>Crown Type</th><th>6m</th><th>12m</th><th>18m</th><th>24m</th><th>36m</th></tr>
<tr><td>GI ZC (%)</td><td>100</td><td>100</td><td>100</td><td>100</td><td>100</td></tr>
<tr><td>PI ZC (%)</td><td>100</td><td>100</td><td>100</td><td>100</td><td>100</td></tr>
<tr><td>GI SSC (%)</td><td>100</td><td>100</td><td>100</td><td>100</td><td>100</td></tr>
<tr><td>PI SSC (%)</td><td>100</td><td>80</td><td>80</td><td>87</td><td>100</td></tr>
</table></td>
<td>The results showed KKZ, and SSC showed no signs of inflammation both scored (100%) in GI. Whereas in PI, KKZ (100%) showed no signs of plaque accumulation as compared to SSC (P=0.047).</td>
</tr>
</table>

Azab et al., [54]	**GI**	1 week, 1, 3, 6, 9, 12, 18, 24, and 36 m	Total number of teeth in each group (N) = 25<br>**(Median) GI**	The results showed that there were no statistically significant differences between GI scores for both cements at different time-points.

**Cement type**	**Baseline**	**1 w**	**1 m**	**3 m**	**6 m**
**Bio-active cement**	0.25	1.13	1.00	0.50	0.38
**Packable GIC**	0.25	1.00	0.50	0.25	0.38
	**9 m**	**12 m**	**18 m**	**24 m**	**36 m**
**Bio-active cement**	0.50	0.25	0.25	0.50	0.25
**Packable GIC**	0.50	0.50	0.38	0.50	0.50

Donly et al., 2018 [55]	**GI**<br>0= Normal<br>1= Mild with slight edema<br>2= Inflammation with edema and bleeding on probing	At 6,12 and 24 m	(see table below)	The results showed that the gingival health appear to be better in NSZ as compared to SSC at 6-month follow up. However, Gingival health appear to be similar in both crowns at 2-year follow up. No crown score C at all evaluation time-points. There were no statistically significant differences (P<0.05) in the GI score for both crowns.

**Total number of teeth in each group (N)**	**6 m N=44**			**12 m N=43**			**24 m N=39**		
**Score**	**0**	**1**	**2**	**0**	**1**	**2**	**0**	**1**	**2**
**NSZ n**	34	9	0	33	10	0	28	8	0
**SSC n**	28	15	0	28	14	0	26	8	0

Hanafi et al., 2021 [56]	**GI:**<br>0= Normal<br>1= Mild inflammation: no BOP<br>2= Moderate inflammation: or BOP	At 1, 3 and 6 m	Total number of (CAD/CAM) ZC (N) = 15<br>Total number of NSZ (N) = 16<br>**GI**	The results showed that CCZC had no changes in gingival appearance, bleeding or gingival recession, and there was improvement in oral health of

	**1m**			**3m**			**6m**		
**Score**	**0**	**1**	**2**	**0**	**1**	**2**	**0**	**1**	**2**

	3= Severe inflammation: a tendency toward spontaneous bleeding.<br>**PI**<br>0= None<br>1= Film at margin<br>2= Moderate<br>3= Abundance amount<br>**BOP (four sites)**<br>0= No bleeding<br>1= Bleeding		**(CAD/CAM) ZC (n)**	0	12	3	0	14	1	0	15	0	anterior crowns. However, there was no statistically significant difference between the two types of crowns at all follow-up phases.
			**NSZ (n)**	0	16	0	0	15	0	0	15	0	
			Total number of (CAD/CAM) ZC (N) = 15<br>Total number of NSZ (N) = 16<br>PI										
			**Score**	**0**	**1**	**2**	**3**	**0**	**1**	**2**	**3**	**0**	
			**(CAD/CAM) ZC (n)**	13	0	2	0	15	0	0	0	15	
			**NSZ (n)**	0	1	15	0	15	0	0	0	15	
			Total number of (CAD/CAM) ZC (N) = 15<br>Total number of NSZ (N) = 16<br>BOP										
			**Score**	**0**	**1**	**0**	**1**	**0**	**1**	**0**	**1**	**0**	
			**(CAD/CAM) ZC (n)**	0	48/60	0	37/60	0	40/60	0	48/60	0	
			**NSZ (n)**	0	49/64	0	37/60	0	41/60	0	49/64	0	

Alaki et al., 2020 [58]	**GI**<br>0= No gingival bleeding<br>1= Bleeding with probe<br>2= Spontaneous bleeding<br>**PI**<br>0= None<br>1= Film at margin<br>2= Moderate<br>3= Abundance amount	At 3, 6 and 12 m	Total number teeth in each group (N) = 60<br>GI (assessed as BOP)				The results showed that the bleeding on probing was greater in RCSC compared to ZC after following up of 3 and 6 months with p-value of (p<0.006) and (p<0.001) respectively. In regards of PI, during 3 and 6 months of follow up the results showed that the teeth covered with ZC had less plaque accumulation compared to teeth covered with RCSC with p-value of (p<0.001) in both groups.
			**Crown type**	**3m**	**6m**	**12m**	
			**ZC n (%)**	24 (40)	0 (100)	0 (100)	
			**RCSC n (%)**	40 (66.7)	28 (46.7)	0 (100)	
			**Total number of teeth in each group (N) = 60**<br>**PI**				

<table>
<tr>
<td></td>
<td></td>
<td></td>
<td>
<table>
<tr><th>Score</th><th>0</th><th>1</th><th>2</th><th>3</th><th>0</th><th>1</th><th>2</th><th>3</th><th>0</th><th>1</th><th>2</th><th>3</th></tr>
<tr><td>ZC n (%)</td><td>20 (33)</td><td>36 (60)</td><td>4 (7)</td><td>0</td><td>47 (80)</td><td>12 (20)</td><td>0</td><td>0</td><td>58 (100)</td><td>0</td><td>0</td><td>0</td></tr>
<tr><td>RCSC n (%)</td><td>4 (7)</td><td>44 (73)</td><td>12 (20)</td><td>0</td><td>24 (40)</td><td>36 (60)</td><td>0</td><td>0</td><td>48 (80)</td><td>12 (20)</td><td>0</td><td>0</td></tr>
</table>
</td>
<td></td>
</tr>
<tr>
<td>Gill et al., 2020 [38]</td>
<td>0= Healthy
1= Red and/or inflamed; no BOP
2= Spontaneous and excessive BOP</td>
<td>At 12 m</td>
<td>
Total teeth number (N) =135
<table>
<tr><th>Score</th><th>0</th><th>1</th><th>2</th><th>Missing n (%)</th></tr>
<tr><td>RCSC n (%)</td><td>27 (56)</td><td>17 (35)</td><td>1 (2)</td><td>3 (6)</td></tr>
<tr><td>PVSSC n (%)</td><td>36 (77)</td><td>11 (23)</td><td>0 (0)</td><td>0 (0)</td></tr>
<tr><td>NSZ n (%)</td><td>33 (83)</td><td>6 (15)</td><td>0 (0)</td><td>1 (3)</td></tr>
<tr><td>Total n (%)</td><td>96 (71)</td><td>34 (25)</td><td>1 (1)</td><td>4 (3)</td></tr>
</table>
</td>
<td>The results showed that NSZ had the highest rate (83%) of healthy gingival tissues when compared to RCSC and PVSSC.</td>
</tr>
<tr>
<td>Sharma et al., 2021 [62]</td>
<td>GI
0= No obvious signs of inflammation
1= Mild marginal gingivitis
2= Moderate marginal gingivitis
3= Severe gingivitis, tissue is very swollen: spontaneous bleeding
PI
0= None
1= Film at margin
2= Moderate
3= Abundance amount</td>
<td>At 12 m</td>
<td>
Total number of RCSC (N) = 17
Total number of ZC (N) = 19
GI
<table>
<tr><th>Score</th><th>0</th><th>1</th><th>2</th></tr>
<tr><td>RCSC n (%)</td><td>27 (56)</td><td>17 (35)</td><td>1 (2)</td></tr>
<tr><td>NSZ n (%)</td><td>33 (83)</td><td>6 (15)</td><td>0 (0)</td></tr>
</table>
Total number of RCSC (N) = 17
Total number of ZC (N) = 19
PI
<table>
<tr><th>Score</th><th>0</th><th>1</th><th>2</th></tr>
<tr><td>RCSC n (%)</td><td>1 (6)</td><td>9 (53)</td><td>7 (41)</td></tr>
<tr><td>NSZ n (%)</td><td>2 (11)</td><td>17 (89)</td><td>0</td></tr>
</table>
</td>
<td>The Results showed that ZCs group had less plaque accumulation and more gingival health than RCSC group. There was a significant increase of plaque accumulation and less gingival health at 12-month follow up in RCSC when compared to ZCs with significant level of (0.013) (0.012) respectively.</td>
</tr>
</table>

Yanover et al., 2020 [63]	0= No obvious signs of inflammation<br>1= Mild marginal gingivitis<br>2= Moderate marginal gingivitis<br>3= Severe gingivitis, tissue is very swollen: spontaneous bleeding	At 6-10.4m, 12.5-17.9m, 18.4-33.8m	Total Number of ZC (N) =131							The results showed that (58%) in 76 teeth with mild gingivitis followed by (35%) in 46 teeth with normal healthy gingiva and 7% in 9 teeth with moderate marginal gingivitis. However, the results obtained that gingival health around all tested ZC was very similar at all intervals of follow up.
			**Score**	**0**	**1**	**2**	**3**			
			**n (%)**	46 (35)	76 (58)	9 (7)	0 (0)			
Talekar et al. 2021 [64]	GI:<br>0= Normal gingiva<br>1= Mild inflammation with slight edema<br>2= inflammation with edema and BOP	At 12 and 18 m	**GI**							The results showed no significant difference between the GFRC and NZS crowns before 12 months period, however, significant differences were found between the tested periods 12 (P>0.05) and 18 months (P<0.001), respectively. The control group experienced severely poor gingival health, while none of the intervention groups experienced this for both periods.
				12 m<br>Total number of GFRC = 30 (%)<br>Total number of NSZ= 31 (%)			18 m<br>Total number of GFRC = 28 (%)<br>Total number of NSZ= 30 (%)			
			**Score**	**0**	**1**	**2**	**0**	**1**	**2**	
			GFRC<br>**n(%)**	24 (80)	5 (17)	1 (3)	20 (71)	6 (22)	2 (7)	
			**NSZ**<br>**n (%)**	29 (93)	2 (7)	0 (0)	28 (93)	2 (7)	0 (0)	
	PI<br>0= no plaque retention<br>1= mild or minimal plaque retention<br>2= Severe plaque retention		**PI**							The results showed that at all periods, **GFRC** crowns scored significant plaque retention (*P*<0.001). Furthermore, only 6% of the tested NSZ crowns were having plaque retention at minor levels after 18 months.
				12 m<br>Total number of GFRC = 30 (%)<br>Total number of NSZ= 31 (%)			18 m<br>Total number of GFRC = 28 (%)<br>Total number of NSZ= 30 (%)			
			**Score**	**0**	**1**	**2**	**0**	**1**	**2**	
			**GFRC**<br>**n (%)**	10 (33)	12 (40)	8 (27)	8 (29)	11 (39)	9 (32)	
			**NSZ**<br>**n (%)**	30 (97)	1 (3)	0 (0)	28 (94)	2 (6)	0 (0)	

PI, Plaque index; GI, Gingival index; ZC, Zirconia crown; C-ZC, Controls for zirconia crowns; SSC, Stainless steel crown; C-SSC, Controls for stainless steel crown; RCSC, Resin composite strip crown; PVSSC, Preveenerd stainless steel crown; ZZC, Zirkiz zirconia crown; EZP, EZ Pedo crown; KKZ, Kinder Krown Zirconia; GIC, Glass ionomer cement; CAD/CAM ZC, Computer-aided manufacturing/Computer-aided modeling zirconia crown; NSZ, NuSmile Zirconia crown; BOP, Bleeding on probing.

Supplemental Table S2. Summary of the included studies' findings about parental satisfaction with zirconia crowns for primary teeth.

Study	Assessed Outcome	Assessment Method	Results							Summary of Results
Holsinger et al., 2016 [44]	1) Parental satisfaction ratings of esthetic characteristics of EZP primary anterior ZCs.<br>2) Parental ratings of the impact of treatment on their children with EZP primary maxillary anterior ZCs	Mean Likert scale rating from 1 (not at all) - 5 (very much)	**Characteristics of EZP**	**Total number of EZP =54**						The results showed that the mean overall satisfaction is 4.6 on a five-point scale. No parents were dissatisfied with the durability, shape, or size of the crowns; one parent reported being dissatisfied with the color of the crowns. Parent's feedback indicated that EZP improved the oral health (83%) and appearance (78%) of their children. Moreover, majority of the parents (83%) indicated that their children did not avoid smiling before treatment.
			**Score**	**1**	**2**	**3**	**4**	**5**	**Mean**	
			**Size: n (%)**	0 (0)	0 (0)	1 (6)	7 (39)	10 (55)	4.5	
			**Shape: n (%)**	0 (0)	0 (0)	3 (17)	4 (22)	11 (61)	4.4	
			**Color: n (%)**	0 (0)	0 (0)	1 (6)	5 (28)	12 (67)	4.6	
			**Parental ratings of the impact of treatment on their children with EZP**							
			**Score**	**1**	**2**	**3**	**4**	**5**	**Mean**	
			**Oral health of child improved after crowns: n (%)**	0 (0)	1 (6)	0 (0)	2(11)	15(83)	4.7	
			**Parents concern about appearance before the crowns: n (%)**	2 (11)	0 (0)	2 (11)	5 (28)	9 (50)	4.1	
			**Child avoided smiling before crowns: n (%)**	15 (83)	0 (0)	2 (11)	1 (6)	0 (0)	1.4	
			**Child smiled after crowns: n (%)**	1 (5.5)	0 (0)	5 (28)	1 (5.5)	11 (61)	4.2	
			**Crowns improved appearance of the child's teeth: n (%)**	0 (0)	0 (0)	1 (6)	3 (16)	14 (78)	4.7	
	1) Parental acceptance		**Total number of teeth in each group (N)=43**							

<table>
<tr>
<td>Salami et al., 2015 [21]</td>
<td>2) Future treatment choice</td>
<td>Likert scale rating of satisfaction from 1 (very unsatisfied) - 5 (very satisfied)</td>
<td>
<table>
<tr><th>Parental Acceptance</th><th>RCSC</th><th>PVSSC</th><th>ZZC</th></tr>
<tr><td>Size: Mean</td><td>4.3</td><td>4</td><td>4.2</td></tr>
<tr><td>Color: Mean</td><td>4.3</td><td>3</td><td>4.1</td></tr>
<tr><td>Shape: Mean</td><td>4.2</td><td>4</td><td>4.3</td></tr>
<tr><td>Durability: Mean</td><td>3.1</td><td>3.9</td><td>4.7</td></tr>
<tr><td>Overall Satisfaction: Mean</td><td>4</td><td>3.8</td><td>4.6</td></tr>
<tr><td>Pain and Discomfort: Mean</td><td>2.2</td><td>1.65</td><td>1.95</td></tr>
</table>
<table>
<tr><th>Future Treatment Choice</th><th>RCSC</th><th>PVSSC</th><th>ZZC</th></tr>
<tr><td>Yes: n (%)</td><td>10 (77)</td><td>10 (83)</td><td>13 (100)</td></tr>
<tr><td>No: n (%)</td><td>3 (23)</td><td>2 (17)</td><td>0 (0)</td></tr>
</table>
</td>
<td>The results showed that ZZC (4.6) scored high overall satisfaction levels than RCSCs (4.0) and PVSSCs (3.8). A significant relationship was found between color of PVSSC ($p=0.003$) and durability of RCSC ($p=0.009$). Moreover, all parents (100%) who chose ZZCs for their children would choose to treat them again with the same crowns.</td>
</tr>
<tr>
<td>Vaishali et al., 2019 [46]</td>
<td>Esthetic acceptability of treatment for anterior teeth with deep caries.</td>
<td>A questionnaire</td>
<td>
<table>
<tr><th colspan="4">Total number of participants = 125</th></tr>
<tr><th>Esthetic acceptability of treatment for anterior teeth with deep caries</th><th>Child</th><th>Mother</th><th>Father</th></tr>
<tr><td>No treatment: (%)</td><td>6</td><td>3</td><td>4</td></tr>
<tr><td>PVSSC: (%)</td><td>13</td><td>9</td><td>8</td></tr>
<tr><td>RCSC: (%)</td><td>16</td><td>12</td><td>10</td></tr>
<tr><td>ZC: (%)</td><td>65</td><td>76</td><td>78</td></tr>
</table>
</td>
<td>The results showed that among both children and their parents ZCs was the most acceptable treatment (65% and 76%) respectively, when used as full-coverage restoration for primary anterior teeth.</td>
</tr>
<tr>
<td>Pani et al., 2016 [47]</td>
<td>Esthetic acceptability of treatment for anterior teeth with deep caries.</td>
<td>A questionnaire</td>
<td>
<table>
<tr><th colspan="4">Total number of participants = 125</th></tr>
<tr><th>Esthetic acceptability of treatment for anterior teeth with deep caries</th><th>Child</th><th>Mother</th><th>Father</th></tr>
<tr><td>No treatment: n (%)</td><td>5 (4.7)</td><td>5 (4.7)</td><td>4 (4.3)</td></tr>
<tr><td>PVSSC: n (%)</td><td>11 (10.3)</td><td>5 (4.7)</td><td>4 (3.7)</td></tr>
</table>
</td>
<td>The results showed that there was no significant difference between parents and their children in the acceptability of any of the treatment modalities ex-</td>
</tr>
</table>

			**RCSC: n (%)**	19 (17.8)	20 (18.7)	16 (18.7)			cept for PVSSC, which were significantly more acceptable to children than their parents.
			**ZC: n (%)**	75 (70.1)	87 (81.3)	85 (79.4)			
Mathew et al., 2020 [49]	Parental satisfaction	Likert type scale from 1 (very unsatisfied) - 5 (very satisfied)	**Comparison of parental satisfaction of SSC and zirconia (%)**						The results showed that only 12 parents (40%) were satisfied with the color of SSCs, whereas with ZCs, all parents (100%) were satisfied with color. A significant statistical difference (P<0.001) was present between both groups. Moreover, all patients (100%) were satisfied with ZCs, but only 16 of the patients (53.3) were satisfied with SSCs. A significant statistical difference (P<0.001) was seen between both groups.
			**Total number of teeth in each group (N)=30**						
			**ZC**	**6 m**	**12 m**	**18 m**	**24 m**	**36 m**	
			**Color**	100	100	100	100	100	
			**Shape**	100	100	100	100	100	
			**Size**	100	100	100	100	100	
			**Retention**	100	100	100	100	100	
			**Durability**	100	100	100	100	100	
			**Overall satisfaction**	100	100	100	100	100	
			**Child satisfaction**	100	100	100	100	100	
			**SSC**						
			**Color**	40	40	40	40	40	
			**Shape**	100	100	100	100	100	
			**Size**	100	100	100	100	100	
			**Retention**	100	100	100	100	100	
			**Durability**	100	100	100	100	100	
			**Overall satisfaction**	100	100	100	100	100	
			**Child satisfaction**	53.3	53.3	53.3	53.3	53.3	
Gill et al., 2020 [38]	Parental satisfaction	A questionnaire	-						The results showed that NSZ had the highest parental esthetic satisfaction when compared to RCSC and PVSSC.
	**1:** Highly satisfied		**Parents Satisfaction**						

Talekar et al. 2021 [64]	**2:** Neutral<br>**3=** Strongly dissatisfied	A questionnaire at two periods: 12 m and 18 m		**12 m**<br>**Total number of GFRC = 30 (%)**<br>**Total number of NSZ= 31 (%)**			**18 m**<br>**Total number of GFRC = 28 (%)**<br>**Total number of NSZ= 30 (%)**			The results showed that among all 31 NSZ, 87% were satisfied for both periods. Almost half of FIGARO (57%) were satisfied at 12 m. The percentage of not satisfied parents increased to 32% at 18 m period with significant differences between both crowns in all periods 12 months (P=0.02) and 18 months (P<0.001).
			**Score**	**1**	**2**	**3**	**1**	**2**	**3**	
			**GFRC n (%)**	19 (57)	8 (34)	3 (9)	17 (68)	0 (0)	11 (32)	
			**NSZ n (%)**	29 (87)	0 (0)	2 (13)	28 (87)	0 (0)	2 (13)	
Yanover et al., 2021 [66]	Parental satisfaction	A questionnaire	-							The results showed that all the tested esthetic crowns have higher parental satisfaction in all aspects; color match, crown contour and crown durability.

EZP, EZ Pedo crown; ZC, Zirconia crown; RCSC, Resin composite strip crown; PVSSC, Preveenerd stainless steel crown; ZZC, Zirkiz zirconia crown; SSC, Stainless steel crown; NSZ, NuSmile Zirconia crown; GFRC, Glass fiber-reinforced composite crown - Figaro Crowns.

Supplemental Table S3. Summary of the included studies' findings about the color stability of zirconia crowns for primary teeth.

**Study**	**Assessment Score**	**Time of Evaluation**	**Results**				**Summary of Results**
Taran et al., 2018 [30]	0=No staining<br>1=Minor staining<br>2=Noticeable staining	At 1,3,6 and 12 m	**Total number of NSZ= 15**				The results showed Minor staining was detected in only one NSZ.
				**Score 0**	**Score 1 at 3 m**	**Score 2**	
			**n (%)**	14 (93)	1 (7)	0 (0)	
Holsinger et al., 2016 [44]	0= No noticeable difference from adjacent teeth<br>1= Slight shade mismatch<br>2= Obvious shade mismatch	2010 – 2014 (Not specified)	**Total number of EZP= 44**				The results showed Shade mismatch was reported in 36% of EZP.
			**Score**	**0**	**1**	**2**	
			**n (%)**	28 (64)	14 (32)	2 (4)	

<table>
<tr>
<td>Mathew et al., 2020 [49]</td>
<td>0=No staining
1=Minor staining
2=Noticeable staining</td>
<td>At 6, 12, 24 and 36 m</td>
<td>
<table>
<tr><th colspan="5">Total number of teeth in each group (N) =30</th></tr>
<tr><th></th><th>Score 0-6 m</th><th>Score 0- 12 m</th><th>Score 0-24 m</th><th>Score 0-36 m</th></tr>
<tr><td>SSC: n (%)</td><td>30 (100)</td><td>30 (100)</td><td>30 (100)</td><td>30 (100)</td></tr>
<tr><td>KKZ: n (%)</td><td>30 (100)</td><td>30 (100)</td><td>30 (100)</td><td>30 (100)</td></tr>
</table>
</td>
<td>The results showed that Both SSC and KKZ reported a successful clinical outcome of 100% with no staining.</td>
</tr>
<tr>
<td>Donly et al., 2018 [55]</td>
<td>Color match (only for ZC):
0= No mismatch
1= Acceptable with a color mismatch
2= Unacceptable with a color mismatch
Staining:
0= No staining
1= Staining that could be polished away
2= Heavy staining that could not be polished away
Marginal discoloration:
0= No discoloration
1= Light discoloration
2= Notable discoloration</td>
<td>At 6,12 and 24 m</td>
<td>
<table>
<tr><th colspan="10">Total number of teeth in each group (N) =43</th></tr>
<tr><th></th><th colspan="3">6 m</th><th colspan="3">12 m</th><th colspan="3">24 m</th></tr>
<tr><th>Score</th><th>0</th><th>1</th><th>2</th><th>0</th><th>1</th><th>2</th><th>0</th><th>1</th><th>2</th></tr>
<tr><td>Color match NSZ: n (%)</td><td>33 (77)</td><td>10 (23)</td><td>0 (0)</td><td>29 (67)</td><td>14 (33)</td><td>0 (0)</td><td>17 (44)</td><td>19 (56)</td><td>0 (0)</td></tr>
<tr><td>Staining NSZ: n (%)</td><td>27 (63)</td><td>16 (37)</td><td>0 (0)</td><td>31 (72)</td><td>12 (28)</td><td>0 (0)</td><td>29 (74)</td><td>7 (18)</td><td>0 (0)</td></tr>
<tr><td>Staining SSC: n (%)</td><td>43 (100)</td><td>0 (0)</td><td>0 (0)</td><td>39 (90)</td><td>2 (10)</td><td>0 (0)</td><td>31 (79)</td><td>1 (2.5)</td><td>0 (0)</td></tr>
<tr><td>Marginal discoloration NSZ: n (%)</td><td>43 (100)</td><td>0 (0)</td><td>0 (0)</td><td>43 (100)</td><td>0 (0)</td><td>0 (0)</td><td>36 (92)</td><td>0 (0)</td><td>0 (0)</td></tr>
<tr><td>Marginal discoloration SSC: n (%)</td><td>43 (100)</td><td>0 (0)</td><td>0 (0)</td><td>42 (98)</td><td>0 (0)</td><td>0 (0)</td><td>34 (87)</td><td>0 (0)</td><td>0 (0)</td></tr>
</table>
</td>
<td>The results showed that there was no statistically significant difference (p<0.05) in the clinical performance (stain resistance, color match and marginal discoloration) at all time-points (6, 12 and 24 months).</td>
</tr>
<tr>
<td>Padmanabh et al., 2021 [57]</td>
<td>Color change:
0= No change
1= change in color</td>
<td>NA</td>
<td>
<table>
<tr><th colspan="3">Total number of teeth in each group (N) = 20</th></tr>
<tr><th>Score</th><th>0</th><th>1</th></tr>
<tr><td>PVSSC n (%)</td><td>20 (100)</td><td>0</td></tr>
<tr><td>SSC n (%)</td><td>20 (100)</td><td>0</td></tr>
<tr><td>ZC n (%)</td><td>20 (100)</td><td>0</td></tr>
</table>
</td>
<td>The results showed that there was no change of the color after sterilization between different types of crowns, in both control and experimental groups.</td>
</tr>
<tr>
<td>Gill et al., 2020 [38]</td>
<td></td>
<td>At 12 m</td>
<td>
<table>
<tr><th colspan="5">Total number of teeth = 135</th></tr>
<tr><th>Score</th><th>0</th><th>1</th><th>2</th><th>Missing n (%)</th></tr>
</table>
</td>
<td>The results showed that (98%) of ZCs completely matched or were</td>
</tr>
</table>

<table>
<tr>
<td></td>
<td>0= Matches completely or within range of shade and translucency
1= Outside range for yellow, yellow/ brown, or grey discoloration
2= Outside range for reason other than yellowing or graying</td>
<td></td>
<td>
<table>
<tr><td>RCSC n (%)</td><td>21 (44)</td><td>23 (48)</td><td>3 (6)</td><td>1 (2)</td></tr>
<tr><td>PVSSC n (%)</td><td>47 (100)</td><td>0 (0)</td><td>0 (0)</td><td>0 (0)</td></tr>
<tr><td>NSZ n (%)</td><td>39 (98)</td><td>0 (0)</td><td>0 (0)</td><td>1 (2)</td></tr>
<tr><td>Total n (%)</td><td>107 (79)</td><td>23 (17)</td><td>3 (2)</td><td>2 (2)</td></tr>
</table>
</td>
<td>within the range of shade and translucency due to the highly polished surface of ZCs which can prevent staining and accumulation of plaque from changing the color.</td>
</tr>
<tr>
<td>Sharma et al., 2021 [62]</td>
<td>0= No noticeable difference from adjacent teeth
1= Slight shade mismatch
2= Obvious shade mismatch</td>
<td>At 12 m</td>
<td>
<table>
<tr><td colspan="4">Total number of RCSC =17
Total number of ZC =19</td></tr>
<tr><td>Score</td><td>0</td><td>1</td><td>2</td></tr>
<tr><td>RCSC n (%)</td><td>6 (35)</td><td>9 (52.9)</td><td>2 (11.7)</td></tr>
<tr><td>NSZ n (%)</td><td>13 (68.4)</td><td>5 (26.3)</td><td>1 (5.2)</td></tr>
</table>
</td>
<td>The results showed that (68.4%) of ZCs had no noticeable change in color when compared to RCSC and After 12 months of follow up but not statistically significant difference (0.168).</td>
</tr>
<tr>
<td>Yanover et al., 2020 [63]</td>
<td>0= No noticeable difference from adjacent teeth
1= Slight shade mismatch
2= Obvious shade mismatch</td>
<td>At 6-10.4m
12.5-17.9m
18.4-33.8m</td>
<td>
<table>
<tr><td colspan="4">Total Number of ZC=131</td></tr>
<tr><td>Score</td><td>0</td><td>1</td><td>2</td></tr>
<tr><td>n(%)</td><td>47(36)</td><td>79(60)</td><td>5(4)</td></tr>
</table>
</td>
<td>The results showed that 47 teeth (36%) scored (0), and 79 teeth (60%) scored (1), and only 5 teeth (4%) scored (2).</td>
</tr>
<tr>
<td>Talekar et al. 2021 [64]</td>
<td>Stain resistance
0: No staining
1: Mild staining could be polished
2= heavy staining cannot be polished</td>
<td>At 12 m and 18 m</td>
<td>
<table>
<tr><td></td><td colspan="3">At 12 m
Total number of GFRC = 30 (%)
Total number of NSZ= 31 (%)</td><td colspan="3">At 18 m
Total number of GFRC = 28 (%) Total number of NSZ= 30 (%)</td></tr>
<tr><td>Score</td><td>0</td><td>1</td><td>2</td><td>0</td><td>1</td><td>2</td></tr>
</table>
</td>
<td>The results no staining in NSZ crown in both periods. On the other hand, 73% of the GFRC group had mild to heavy staining in the first 12 m period, this percentage increased to 86% at 18 m period. Significant differences of (P <0.001)</td>
</tr>
</table>

			**GFRC n(%)**	8 (27)	12 (40)	10 (33)	4 (14)	14 (50)	10 (36)	between both groups at the two periods.
			**NSZ n (%)**	31 (100)	0 (0)	0 (0)	30 (100)	0 (0)	0 (0)	
	**Color match**<br>**0=** No noticeable difference from adjacent teeth<br>**1=** Slight shade mismatch<br>**2=** Obvious shade mismatch	Upon crown selection		**Total number of GFRC = 33 (%)**<br>**Total number of NSZ= 33 (%)**						
			**Score**	**0**		**1**		**2**		All the NSZ were matched the natural color of the adjacent teeth and half of the GFRC had a notable mismatching of the color.
			**GFRC n(%)**	19 (58)		11 (33)		3 (9)		
			**NSZ n (%)**	30 (100)		0		0		

Abbreviations: NSZ, NuSmile Zirconia crown; EZP, EZ Pedo crown; SSC, Stainless steel crown; KKZ, Kinder Krown Zirconia crown; ZC, Zirconia crown; PVSSC, Preveenerd stainless steel crown; RCSC, Resin composite strip crown; NA, Not Applicable; GFRC, Glass fiber-reinforced composite crown - Figaro Crowns; DCR, Direct composite restoration.

Supplemental Table S4. Summary of the included studies' findings about the retention of zirconia crowns for primary teeth.

**Study**	**Assessment Score/Method**	**Time of Evaluation**	**Results**					**Summary of Results**
Taran et al., 2018 [30]	0= Present (Retained)<br>1= Absent (Lost)	At 1, 3, 6 and 12 m	**Total number of teeth in each group (N)=15**					The results showed that SSC had a higher retention rate than NSZ.
				**Score 0**	**Score 1**			
			**NSZ: n (%)**	13 (87)	2 (13)			
			**SSC: n (%)**	15 (100)	0 (0)			
Walia et al., 2014 [27]	0= Normal<br>1= Small but noticeable area of loss of material<br>2= Large loss of material<br>3= Complete loss	At 6 m	**Total number of RCSC =36**<br>**Total number of PVSSC =37**<br>**Total number of ZZC =38**					The results showed that ZZC reported a higher retention rate than RCSC and PVSSC crowns. There was a statistically significant difference between RCSC and PVSSC (p=0.04)
				**Score 0**	**Score 1**	**Score 2**	**Score 3**	
			**RCSC: n (%)**	28 (78)	2 (5)	0 (0)	6 (17)	
			**PVSSC: n (%)**	35 (95)	2 (5)	0 (0)	0 (0)	

			**ZZC: n (%)**	38 (100)	0 (0)	0 (0)	0 (0)		and between RCSC and ZZC (p=0.02).
Holsinger et al., 2016 [44]	0= Intact<br>1= Chipped/small but noticeable area of loss of material<br>2= Large loss of material<br>3 = Complete loss	(Retrospective study)<br>2010 – 2014	**Total number of EZP = 46**						The results showed that 96% of EZP were retained and only 4% were lost.
				**Score 0**	**Score 1**	**Score 2**	**Score 3**		
			**n (%)**	44 (96)	0 (0)	0 (0)	2 (4)		
Jing et al., 2019 [45]	Universal testing machine	NA	**Total number of SEC = 46**						The result showed that there were significantly lower MPa between the 1 mm group and the rest of groups 2,3 and 4mm (P<0.001). There were no significant differences in MPa among the 2,3 and 4mm groups (P>0.05) and there was no difference in retention between the TC and NTC.
			**OCH Mean**	**1 mm TC**	**2 mm TC**	**3 mm TC**	**4 mm TC**	**3 mm NTC**	
			Mean (Newtons)	116.0	251.4	319.3	431.5	298.0	
			Mean (MPa)	1.7	2.9	3.1	3.3	2.7	
			SD	1.0	1.4	0.7	0.6	0.8	
			Range (MPa)	2.9	4.3	2.1	2.3	2.4	
Mathew et al., 2020 [49]	0= Present (Retained)<br>1= Absent (Lost)	At 6, 12, 24, and 36 m	**Total number of teeth in each group (N)=30**						The results showed that Both SSC and KKZ reported a 100% retention.
				**Score 0- 6 m**	**Score 0- 12 m**	**Score 0- 24 m**	**Score 0- 36 m**		
			**SSC: n (%)**	30 (100)	30 (100)	30 (100)	30 (100)		
			**KKZ: n (%)**	30 (100)	30 (100)	30 (100)	30 (100)		
Azab et al., [54]	By visual inspection and probe to detect debonded crowns	At 1 week, 1, 3, 6, 9, 12, 18, 24, and 36 m	**Retained Crowns**						The results showed that there were statistically significant more retained and less debonded crowns for packable GI group compared to bio-
			**Cement type**	**N=25 1 w**	**N=24 1 m**	**N=22 3 m**	**N=21 6 m**	**N=21 9 m**	
			**Bio-active cement**	23 (92)	19 (79)	13 (62)	10 (48)	7 (33.3)	

<table>
<tr>
<td></td>
<td></td>
<td></td>
<td>
<table>
<tr><td>Packable GIC</td><td>24 (96)</td><td>23 (95)</td><td>21 (95)</td><td>20 (95)</td><td>20 (95)</td></tr>
<tr><td></td><td>N=21
12 m</td><td>N=21
18 m</td><td>N=21
24 m</td><td colspan="2">N=19 (Bioactive Cement)
N=17 (Packable GIC)
36 m</td></tr>
<tr><td>Bioactive cement</td><td>7 (33.3)</td><td>7 (33.3)</td><td>7 (33.3)</td><td colspan="2">4 (21)</td></tr>
<tr><td>Packable GIC</td><td>20 (95)</td><td>20 (95)</td><td>20 (95)</td><td colspan="2">14 (82)</td></tr>
</table>
</td>
<td>active cement group at 3–36-month follow-ups.</td>
</tr>
<tr>
<td>Al Shahawy et al., 2016 [37]</td>
<td>Clinically and radiographically</td>
<td>At 3, 6, 12, 18 and 24 m</td>
<td>
<table>
<tr><td colspan="6">Total number of NSZ =86</td></tr>
<tr><td></td><td>3 m</td><td>6 m</td><td>12 m</td><td>18 m</td><td>24 m</td></tr>
<tr><td>n (%)</td><td>86 (100)</td><td>85 (98)</td><td>77 (96)</td><td>68 (92)</td><td>61 (91)</td></tr>
</table>
</td>
<td>The results showed that (91%) of NSZ were retained and only (9%) dislodged after 24-months. The overall survival of the restorations was 95.3% after 12 months and 80.2% after 24 months.</td>
</tr>
<tr>
<td>Gill et al., 2020 [38]</td>
<td>0= Intact
1= Partially missing
2= Missing-some cement remaining on both tooth and crown interior</td>
<td>At 12 m</td>
<td>
<table>
<tr><td colspan="5">Total number of teeth = 135</td></tr>
<tr><td>Score</td><td>0</td><td>1</td><td>2</td><td>Missing n (%)</td></tr>
<tr><td>RCSC n (%)</td><td>38 (79)</td><td>6 (13)</td><td>3 (6)</td><td>1 (2)</td></tr>
<tr><td>PVSSC n (%)</td><td>47 (100)</td><td>0 (0)</td><td>0 (0)</td><td>0 (0)</td></tr>
<tr><td>NSZ n (%)</td><td>38 (95)</td><td>1 (3)</td><td>0 (0)</td><td>1 (3)</td></tr>
<tr><td>Total n (%)</td><td>123 (91)</td><td>7 (5)</td><td>3 (2)</td><td>2 (2)</td></tr>
</table>
</td>
<td>The results showed that (95%) of NSZ were intact after 12 months follow up.</td>
</tr>
<tr>
<td>Alaki et al., 2020 [58]</td>
<td>0= Crown appears normal
1= Small but noticeable area of loss of material
2= Large loss of crown material</td>
<td>At 3, 6 and 12 m</td>
<td>
<table>
<tr><td colspan="13">Total number of teeth in each group (N) =60</td></tr>
<tr><td></td><td colspan="4">3m</td><td colspan="4">6m</td><td colspan="4">12m</td></tr>
<tr><td>Score</td><td>0</td><td>1</td><td>2</td><td>3</td><td>0</td><td>1</td><td>2</td><td>3</td><td>0</td><td>1</td><td>2</td><td>3</td></tr>
<tr><td>RCSC n (%)</td><td>60 (100)</td><td>0</td><td>0</td><td>0</td><td>52 (87)</td><td>7 (12)</td><td>1 (1)</td><td>0</td><td>37 (62)</td><td>18 (30)</td><td>5 (8)</td><td>0</td></tr>
</table>
</td>
<td>The results showed that there were no crown failures up to 3 months follow up in both groups, in ZC group 2 crowns had lost due to trauma in 6 and 12 follow up, while failure was noticed more in RCSC group in both</td>
</tr>
</table>

	3= Complete loss of crown		**ZC n (%)**	60 (100)	0	0	0	59 (98)	0	0	1 (2)	58 (98)	0	0	1 (2)	intervals of follow up 6 and 12 months.

Alhissan et al., 2021 [59]	0= No clinical failure<br>1= Debonding without complications<br>2= Debonding with complications<br>3= Failure without debonding	At 24 m	**Total number of ZC =70**			The results showed that out of the 70 ZCs studied over 24 months, 14 crowns had failed, giving an overall success rate of 80%. The success rate of crowns with pulp treatment was significantly lower (76%) than crowns without pulp treatment (90%).
			**Score**	**With pulp therapy**	**Without pulp therapy**	
			**0**	18	38	
			**1**	2	8	
			**2**	0	3	
			**3**	0	1	
			**Total**	20	50	

Sharma et al., 2021 [62]	0= Crown appears normal:<br>1= Small but noticeable area of loss of material<br>2= Large loss of crown material<br>3= Complete loss of crown	At 12 m	**Total number of RCSC =19**<br>**Total number of ZC =20**					The results showed that restoration failure was significantly higher in RCSC group when compared to ZCs with significant level of (0.0001).
			Score	0	1	2	3	
			RCSC n (%)	5 (26.3)	11 (57.8)	1 (5.2)	2 (10.5)	
			ZC n (%)	19 (95)	0	0	1 (5)	

Talekar et al. 2021 [64]	0= Intact or present<br>1= Chipped / loss of material<br>2= Complete loss of the crown.	At 12 m and 18 m		**At 12 m**<br>**Total number of GFRC = 33 (%)**<br>**Total number of NSZ= 33 (%)**			**At 18 m**<br>**Total number of GFRC = 29 (%)**<br>**Total number of NSZ= 30 (%)**			The results showed a significant difference (P<0.05) between the two periods. Only two of the NSZ crowns were dislodged at 12 m period with no chipping or more additional losses later. Half of GFRC crowns were damaged and the losses were double of NSZ at 18 m period.
			**Score**	**0**	**1**	**2**	**0**	**1**	**2**	
			**GFRC n (%)**	23 (70)	7 (21)	3 (9)	15 (52)	13 (45)	1 (3)	
			**NSZ n (%)**	31 (94)	0 (0)	2 (6)	30 (100)	0	0	

Walia et al 2021[67]	Universal testing machine	NA	**Total number of each group (N)= 24**					The results showed that all the esthetic tested crowns have better retention with BioCem and FujiCEM® 2 with no significant difference between them. However, the Ketac Cem Maxicap cement was significantly lower and less retentive (P= 0.002).
			**Cement type**	**NSZ**	**CCZ**	**SEC**	**KKZ**	
			**BioCem: n (SD)**	112 (17)	110 (15)	130 (23)	117 (22)	
			**FujiCEM®: n (SD)**	106 (14)	129 (25)	111 (17)	114 (21)	
			**Ketac Cem Maxicap: n (SD)**	44 (10)	31 (10)	27 (12)	20 (9)	

Abbreviation: MPa, Megapascal; OCH, Occluso-cervical heights; TC, Thermocycling; NTC, Non-thermocycling “control group”; SD, Standard deviation; SSC, Stainless steel crown; KKZ, Kinder Krown Zirconia crown; GIC, Glass ionomer cement; RCSC, Resin composite strip crown; PVSSC, Preveenerd stainless steel crown; NSZ, NuSmile Zirconia crown; EZP, EZ Pedo crown; ZZC, Zirkiz zirconia crown; GFRC, Glass fiber-reinforced composite crown-Figaro Crowns.

Supplemental Table S5. Summary of the included studies’ findings about the fracture resistance of zirconia crowns for primary teeth.

**Study**	**Assessment Score/Method**	**Time of Evaluation**	**Results**				**Summary of Results**
Tara et al., 2018 [30]	0= Intact<br>1= <50% surface chipped<br>2= >50% surface chipped	At 1, 3, 6 and 12 m.	**Total number of NSZ= 15**				The results showed that only one NSZ has fractured at the 12-month follow-up.
				**Score 0**	**Score 1**	**Score 2 at 12 m**	
			**n (%)**	14 (93)	0 (0)	1 (7)	
Kist et al ., 2019 [42]	Universal testing machine	NA	**Total number of crowns= 85**				The results showed that among the prefabricated ZCs before aging, NZS had the highest fracture loads, followed by KKZ and SEC. After aging, results showed that KKZ scored the highest fracture loads among all prefabricated ZCs. However, the CAD/CAM crowns showed Significantly higher fracture loads than prefabricated ZCs. The PVSSC showed the highest fracture loads when compared with all types of ZCs, both with and without aging.
			**SEC: Mean (SD)**	893 (223)			
			**KKZ: Mean (SD)**	1570 (368)			
			**NSZ: Mean (SD)**	1582 (638)			
			**CAD/CAM ZC: Mean (SD)**	2444 (360)			
			**PVSSC: Mean (SD)**	6251 (1170)			

Al shobber et al., 2017 [50]	Universal testing machine	NA	**Total number of teeth in each group (N)=16**					The results showed that NZS scored the highest fracture loads. Whereas CCP, showed the lowest forces required for fracture of the crown. Both NSZ and CCZ showed statistically significant difference in forces required to fracture the crowns when compared to PVSSC (p<0.05).
			**CCP: Mean (SD)**	415.58 (12.28)				
			**CCZ: Mean (SD)**	751.43 (102.1)				
			**NSZ: Mean (SD)**	646.68 (229.62)				
			**PVSSC: Mean (SD)**	482.37 (76.94)				
El Makawi et al., 2019 [43]	Universal testing machine	NA	**Total number of teeth in each group (N)=10**					The results showed that NSZ scored high mean and SD values. While LDE scored lower mean and SD with values with a statistically significant difference between them (p=0.0001).
			**NSZ: Mean (SD)**	1420.893 (308.39)				
			**LDE: Mean (SD)**	854.427 (130.52)				
Townsend et al., 2014 [53]	Universal testing machine	NA	**Total number of teeth in each group (N)=20**					The results showed that the forces required to fracture EZP was significantly higher than NSZ and KKZ (P<0.001). In addition, NSW required significantly higher forces to be fractured more than all three ZCs (P<0.001).
			**EZP: Mean (SD)**	1091(146.5)				
			**KKZ: Mean (SD)**	576 (132.3)				
			**NSZ: Mean (SD)**	691 (113.3)				
			**PVSSC: Mean (SD)**	1937(545.5)				
Azab et al., [54]	Visual inspection to detect incomplete fracture lines or complete fractures with loss of crown material	At 1 week, 1, 3, 6, 9, 12, 18, 24, and 36 m	Zero fractured crowns at all follow-up time-points.					The results showed that there were no fractured crowns noted for both types of cements.
Padmanabh et al., 2021 [57]	0= No fracture<br>1= Cracks detected visually and manually less than 1/3 of surface<br>2= Cracks detected visually and manually from 1/3 to 1/2 of surface<br>3= Cracks detected visually and manually more than 1/2 of surface	NA	**Total number of teeth in each group (N)=20**					The results showed that there were no fractures noted after sterilization between different types of crowns in all control and experimental groups.
			**Score**	**0**	**1**	**2**	**3**	
			**PVSSC n (%)**	20 (100%)	0	0	0	
			**SSC n (%)**	20 (100%)	0	0	0	
			**ZC n (%)**	20 (100%)	0	0	0	
Talekar et al. 2021	0= intact occlusal surface			**At 12 m**		**At 18 m**		

<table>
<tr>
<td>[64]</td>
<td>1= Wear of the occlusal surface without tooth surface exposure
2= Wear of occlusal
surface with tooth surface exposure</td>
<td>At 12 m and 18 m</td>
<td>
<table>
<tr><td></td><td colspan="3">Total number of GFRC = 30 (%)
Total number of NSZ= 31 (%)</td><td colspan="3">Total number of GFRC = 28 (%)
Total number of NSZ= 30 (%)</td></tr>
<tr><td>Score</td><td>0</td><td>1</td><td>2</td><td>0</td><td>1</td><td>2</td></tr>
<tr><td>GFRC n(%)</td><td>23 (70)</td><td>7 (21)</td><td>3 (9)</td><td>11 (40)</td><td>10 (35)</td><td>7 (25)</td></tr>
<tr><td>NSZ n (%)</td><td>31 (100)</td><td>0 (0)</td><td>0 (0)</td><td>31 (100)</td><td>0 (0)</td><td>0 (0)</td></tr>
</table>
</td>
<td>The results showed a significant difference (P<0.001) between the two periods. No signs of crown wear were noticed in NSZ, and they were 100% intact at both periods. Almost a third of GFRC crowns experienced occlusal wear with complete tooth surface exposure.</td>
</tr>
<tr>
<td>Lin et al. 2021 [65]</td>
<td>Universal testing machine</td>
<td>NA</td>
<td>
<table>
<tr><td colspan="2">Total number of teeth in each group (N)=15</td></tr>
<tr><td>EZP with Type I GIC: Mean (SD)</td><td>410.9 (79.5)</td></tr>
<tr><td>EZP with self-adhesive resin cement: Mean (SD)</td><td>420.5 (57.8)</td></tr>
<tr><td>Polycarbonate crowns: Mean (SD)</td><td>330 (85.6)</td></tr>
<tr><td>RCSC: Mean (SD)</td><td>268.4 (25.8)</td></tr>
</table>
</td>
<td>The results showed the mean and SD of the required forces of fracture in Newton. The required forces for fracture of EZP – regardless of cement type- are significantly higher than those of the other tested crowns (P= <.0001).</td>
</tr>
<tr>
<td>Sabbah et al., 2020 [68]</td>
<td>Universal testing machine</td>
<td>NA</td>
<td>
<table>
<tr><td colspan="2">Total number of teeth in each group (N) = 6</td></tr>
<tr><td>Crown Type</td><td>Mean (SD)</td></tr>
<tr><td>NSZ</td><td>1229 (192.6)</td></tr>
<tr><td>NCCE</td><td>845,4 (51.5)</td></tr>
</table>
</td>
<td>The Results showed higher mean fracture resistance in ZC compared to endocrown with statistically significant difference (p-value = 0.001).</td>
</tr>
</table>

Mohn et al., 2021[69]	Thermomechanical loading	NA	-	The results showed that (CAD/CAM) ZC reported the lowest wear resistance among other groups. Whereas RCSC showed significantly lower wear depth compared to gold standard. However, there was no sign of wear on the surface of NSZ. On other hand, KKZ showed 119-micrometervertical wear along with microcrack signs.<br>Furthermore, (CAD/CAM) ZC exhibit significantly lower antagonist wear than SSC (p < .05).
Abbreviation: EZP, EZ Pedo crown; KKZ, Kinder Krown Zirconia crown; NSZ, NuSmile Zirconia crown; NSW, Nusmile Signature crown; ZC, Zirconia crown; NA, Not applicable; SEC, Spring EZ crown; CAD/CAM, Computer-aided manufacturing/Computer-aided modeling; CCZC, CAD/CAM zirconia crown; PVSSC, Preveenerd stainless steel crown; SD, Standard deviation; CCZ, Cheng crown zirconia; CCP, Cheng crown preveenred; LDE, Lithium disilicate endocrown; SSC, Stainless steel crown; GFRC, Glass fiber-reinforced composite crown - Figaro Crowns; NCCE, Nano-Ceramic Compo-site Endo-crowns.				

Supplemental Table S6. Summary of the included studies' findings about the marginal integrity of zirconia crowns for primary teeth.

**Study**	**Assessment Method**	**Time of Evaluation**	**Results**			**Summary of Results**
Holsinger et al., 2016 [44]	Visible inspection and tactile examination with an explorer<br>0= Closed margin<br>1= Open margin	(Retrospective study)<br>2010 – 2014	**Total number of EZP (N) =44**			The results showed that majority of EZP (86%) had closed margins, and few (14%) had open margins.
			**Score**	**0**	**1**	
			**EZP n(%)**	38 (86)	6 (14)	
AlHaj etal., 2019 [52]	Thirteen measurements were made at 13 different sites on the digital photographs of the sectioned surfaces of the specimens using	NA	**Total number of teeth in each group (N) = 15**<br>**Mean (SD)**			The results showed that NSZ cemented with resin cement had significantly lower internal gap width mean than PVSSCs and SSCs cemented with resin cement (P<0.001). In addition, the difference between NSZ, PVSSC and SSC and luting
			**Gap**	**Crown type**	**Luting cement**	
				**SSC**	**GIC**	
			**Marginal**	0.35 (0.17)	0.38 (0.16)	
			**Internal**	0.34 (0.12)	0.37 (0.09)	

<table>
<tr>
<td></td>
<td>a modified method to that adopted by Korkut et al.</td>
<td></td>
<td>
<table>
<tr><td></td><td>PVSSCs</td><td>RMGIC</td></tr>
<tr><td>Marginal</td><td>0.34 (0.22)</td><td>0.26 (0.18)</td></tr>
<tr><td>Internal</td><td>0.38 (0.12)</td><td>0.26 (0.11)</td></tr>
<tr><td></td><td>NSZ</td><td>Resin Cement</td></tr>
<tr><td>Marginal</td><td>0.17 (0.09)</td><td>0.24 (0.20)</td></tr>
<tr><td>Internal</td><td>0.19 (0.12)</td><td>0.29 (0.20)</td></tr>
</table>
</td>
<td>cement used, was not significant regarding the width of the marginal gap (P>0.05).</td>
</tr>
<tr>
<td>Donly et al., 2018 [55]</td>
<td>A= Clinically ideal, with no ditching or gap
B= Clinically acceptable, with evidence of ditching or gap
C= Clinically unacceptable, with mobility, making crown replacement necessary.</td>
<td>At 6, 12, and 24 m</td>
<td>
<table>
<tr><td>Total number of teeth in each group (N)</td><td colspan="3">6m
N=44</td><td colspan="3">12m
N=43</td><td colspan="3">24m
N=39</td></tr>
<tr><td>Score</td><td>A</td><td>B</td><td>C</td><td>A</td><td>B</td><td>C</td><td>A</td><td>B</td><td>C</td></tr>
<tr><td>NSZ (n)</td><td>39</td><td>4</td><td>0</td><td>37</td><td>6</td><td>0</td><td>33</td><td>3</td><td>0</td></tr>
<tr><td>SSC (n)</td><td>42</td><td>1</td><td>0</td><td>41</td><td>1</td><td>0</td><td>33</td><td>1</td><td>0</td></tr>
</table>
</td>
<td>The results showed that the majority NSZ scored A except 13 crowns scored B, 4 of them at 6 months follow-up, 6 of them at 12 month and only 3 at 24 months. Whereas in SCC, almost all crowns scored A and only 1 scored B, at 6, 12- and 24-months follow-up.</td>
</tr>
<tr>
<td>Alaki et al., 2020 [58]</td>
<td>0= Close marginal adaptation
1= No detectable margin
2= Detectable margin</td>
<td>At 3, 6 and 12 m</td>
<td>-</td>
<td>The results showed that 11.7% of the RCSC group had small but noticeable area of material marginal loss.</td>
</tr>
<tr>
<td>Gill et al., 2020 [38]</td>
<td>0= No discrepancies detected with explorer
1= Detectable discrepancies but clinically acceptable
2= Detectable discrepancies, replacement required</td>
<td>At 12 m</td>
<td>
<table>
<tr><td colspan="5">Total number of teeth (N) = 135</td></tr>
<tr><td>Score</td><td>0</td><td>1</td><td>2</td><td>Missing n (%)</td></tr>
<tr><td>RCSC n (%)</td><td>33 (69)</td><td>11 (23)</td><td>3 (6)</td><td>1 (2)</td></tr>
<tr><td>PVSSC n (%)</td><td>46 (98)</td><td>1 (2)</td><td>0 (0)</td><td>0 (0)</td></tr>
<tr><td>NSZ n (%)</td><td>37 (93)</td><td>2 (5)</td><td>0 (0)</td><td>1 (3)</td></tr>
<tr><td>Total n (%)</td><td>116 (86)</td><td>14 (10)</td><td>3 (2)</td><td>2 (2)</td></tr>
</table>
</td>
<td>The results showed that NSZ had the second highest rate (93%) when measuring the marginal adaptation.</td>
</tr>
<tr>
<td>Nischal et al., 2020 [60]</td>
<td>Alpha criteria rating system.</td>
<td>At 3, 6 and 9 m</td>
<td>
<table>
<tr><td colspan="4">Total number of teeth in each group (N) = 15</td></tr>
<tr><td></td><td>3m</td><td>6m</td><td>9m</td></tr>
</table>
</td>
<td></td>
</tr>
</table>

			**Score**	**A**	**B**	**C**	**D**	**A**	**B**	**C**	**D**	**A**	**B**	**C**	**D**	The results showed that ZCs had no change in the marginal integrity after 9 months of recall.
			**RCSC n (%)**	15 (100)	0	0	0	12 (80)	3 (20)	0	0	9 (60)	3 (20)	1 (7)	2 (13)	
			**ZC n (%)**	15 (100)	0	0	0	15 (100)	0	0	0	15 (100)	0	0	0	
			**Luxa Crown n (%)**	15 (100)	0	0	0	15 (100)	0	0	0	11 (87)	1 (7)	1 (7)	2 (13)	
Yanover et al., 2020 [63]	0= Continuous with the contour of the crown<br>1= Slight overhang or under-contour present<br>2= Large defects noted	At 6-10.4m, 12.5-17.9m, 18.4-33.8m	**Total Number of ZC (N) =1 31**													The results showed that the majority of the ZCs were nicely adapted 82.4% with only 13.7% of the crowns were slight over-hanged or under-contoured and 3.9% were found with large defects.
			**Score**	**0**	**1**	**2**										
			**n (%)**	107 (82.4)	18 (13.7)	6 (3.9)										
Möhn et al., 2021 [69]	Thermomechanical loading	N/A	-													The results showed that (CAD/CAM) ZC reported a significantly higher marginal adaptation compared to other crowns. Whereas ZC cemented with GI cement revealed lowest continuity in the margins.

Abbreviation: EZP, EZ Pedo crown; NA, Not applicable; SD, Standard deviation; SSC, Stainless steel crown; PVSSC, Preveenerd stainless steel crown; GIC, Glass ionomer cement; RMGIC, Resin modified glass ionomer cement; NSZ, NuSmile Zirconia crown; RCSC, Resin composite strip crown; ZC, Zirconia crown.

Supplemental Table S7. Summary of the included studies' findings about the surface roughness of zirconia crowns for primary teeth.

Study	Assessment Method	Results					Summary of Results
Walia et al., 2019 [41]	Using a mechanical surface roughness tester/ profilometer	**Total number of teeth in each group (N) = 10**<br>**Surface roughness Mean (SD)**					The results showed that there were significantly different surface roughness and roughness depth scores for KKZ first and second molar when compared to CCZ, SEC and NSZ for occlusal surfaces (p=0.013). A similar result was observed for occlusal edges for first and second primary molars (p = 0.013).
		**Crown type**	**Occlusal surface First molar**	**Occlusal surface Second molar**	**Occlusal edge (buccal cusps) First molar**	**Occlusal edge (buccal cusps) Second molar**	
		**CCZ**	0.34 (0.07)	0.36 (0.04)	0.30 (0.04)	0.28 (0.02)	
		**SEC**	0.39 (0.09)	0.45 (0.07)	0.41 (0.08)	0.36 (0.03)	
		**NSZ**	0.37 (0.06)	0.40 (0.06)	0.40 (0.06)	0.35 (0.05)	
		**KKZ**	0.70 (0.03)	0.91 (0.09)	1.04 (0.26)	0.60 (0.12)	
		**Roughness depth Mean (SD)**					
			**Occlusal surface First molar**	**Occlusal surface Second molar**	**Occlusal edge (buccal cusps) First molar**	**Occlusal edge (buccal cusps) Second molar**	
		**CCZ**	1.10 (0.08)	0.86 (0.12)	1.03 (0.09)	1.03 (0.09)	
		**SEC**	1.20 (0.28)	1.30 (0.63)	1.30 (0.16)	1.56 (0.44)	
		**NSZ**	1.53 (0.16)	0.90 (0.08)	1.10 (0.01)	1.16 (0.12)	
		**KKZ**	2.23 (0.28)	2.23 (0.28)	3.5 (0.99)	2.66 (0.36)	
Theriot et al., 2017 [51]	Using a mechanical surface roughness tester/ profilometer	**Total number of teeth in each group (N) = 20**<br>**Surface roughness Mean (SD)**					The results showed that the lingual surfaces of NSZ had a statistically significant lower mean of surface roughness than the lingual surfaces of SEC and KKZ (P<0.001).
			**Labial crown surface**	**Lingual crown surface**			
		**NSZ**	2.8 (0.3)	1.9 (0.5)			

<table>
<tr><td rowspan="2"></td><td rowspan="2"></td><td colspan="5">SEC</td><td colspan="4">2.5 (0.51)</td><td colspan="4">3.7 (0.8)</td><td rowspan="2">Whereas in the labial surfaces of KKZ, results showed a statistically significant higher mean of surface roughness than the labial surfaces of NSZ and SEC (P<0.001).</td></tr>
<tr><td colspan="5">KKZ</td><td colspan="4">4.3 (0.61)</td><td colspan="4">3.2 (0.6)</td></tr>
<tr><td rowspan="4">Donly et al., 2018 [55]</td><td rowspan="4">A= Ideal, with a smooth crown surface
B= Clinically acceptable, with a slightly rough
C= Clinically unacceptable, with a crown fracture</td><td>Total number of teeth in each group (N)</td><td colspan="4">6m
N=43</td><td colspan="4">12m
N=43</td><td colspan="4">24m
N=39</td><td rowspan="4">The results showed that NSZ has scored A (clinically ideal) in all time points 6, 12 and 24 months except two crowns scored B, one crown after 6 months and the other after 24 months with no crown scored C. Whereas in SSC, all crowns scored A at all evaluation time-points. There were no statistically significant differences between both crowns (P<0.05).</td></tr>
<tr><td>Score</td><td>A</td><td>B</td><td colspan="2">C</td><td>A</td><td>B</td><td colspan="2">C</td><td>A</td><td>B</td><td colspan="2">C</td></tr>
<tr><td>NSZ (n)</td><td>42</td><td>1</td><td colspan="2">0</td><td>43</td><td>0</td><td colspan="2">0</td><td>35</td><td>1</td><td colspan="2">0</td></tr>
<tr><td>SSC (n)</td><td>43</td><td>0</td><td colspan="2">0</td><td>42</td><td>0</td><td colspan="2">0</td><td>34</td><td>0</td><td colspan="2">0</td></tr>
<tr><td rowspan="6">Nischal et al., 2020 [60]</td><td rowspan="6">The Modified United States Public Health Service (USPHS) criteria</td><td colspan="13">Total number of teeth in each group (N) = 15</td><td rowspan="6">The results showed that at 9-month follow up, the surface texture of ZCs did not change.</td></tr>
<tr><td></td><td colspan="4">3m</td><td colspan="4">6m</td><td colspan="4">9m</td></tr>
<tr><td>Score</td><td>A</td><td>B</td><td>C</td><td>D</td><td>A</td><td>B</td><td>C</td><td>D</td><td>A</td><td>B</td><td>C</td><td>D</td></tr>
<tr><td>RCSC n (%)</td><td>15 (100)</td><td>0</td><td>0</td><td>0</td><td>15 (100)</td><td>0</td><td>0</td><td>0</td><td>12 (80)</td><td>3 (20)</td><td>0</td><td>0</td></tr>
<tr><td>ZC n (%)</td><td>15 (100)</td><td>0</td><td>0</td><td>0</td><td>15 (100)</td><td>0</td><td>0</td><td>0</td><td>15 (100)</td><td>0</td><td>0</td><td>0</td></tr>
<tr><td>Luxa Crown n (%)</td><td>15 (100)</td><td>0</td><td>0</td><td>0</td><td>15 (100)</td><td>0</td><td>0</td><td>0</td><td>13 (87)</td><td>2 (13)</td><td>0</td><td>0</td></tr>
<tr><td colspan="16">Abbreviation: SD, Standard deviation; CCZ, Cheng crown zirconia; SEC, Spring EZ crown; SSC, Stainless steel crown; NSZ, NuSmile Zirconia crown; KKZ, Kinder Krown Zirconia crown; RCSC, Resin composite strip crown; ZC, Zirconia crown.</td></tr>
</table>

Supplemental Table S8. Summary of the included studies' findings about recurrent caries with zirconia crowns for primary teeth.

<table>
<tr><th>Study</th><th>Assessment Score</th><th>Time of Evaluation</th><th colspan="7">Results</th><th>Summary of Results</th></tr>
<tr><td rowspan="3">Holsinger et al., 2016 [44]</td><td rowspan="3">0= No caries
1=Recurrent caries</td><td rowspan="3">(Retrospective study)
2010 – 2014</td><td colspan="7">Total number of EZP (N) = 44</td><td rowspan="3">The results showed that EZP did not report recurrent caries.</td></tr>
<tr><td></td><td colspan="3">Score 0</td><td colspan="3">Score 1</td></tr>
<tr><td>n (%)</td><td colspan="3">44 (100)</td><td colspan="3">0 (0)</td></tr>
<tr><td rowspan="4">Donly et al., 2018 [55]</td><td rowspan="4">0= No caries
1=Recurrent caries</td><td rowspan="4">At 6,12 and 24 m</td><td>Total number of teeth in each group (N)</td><td colspan="2">6 m
N=43</td><td colspan="2">12 m
N=43</td><td colspan="2">24 m
N=39</td><td rowspan="4">The results indicated that both NSZ and SSC showed no recurrent caries.</td></tr>
<tr><td>Score</td><td>0</td><td>1</td><td>0</td><td>1</td><td>0</td><td>1</td></tr>
<tr><td>NSZ: n</td><td>43</td><td>0</td><td>43</td><td>0</td><td>36</td><td>0</td></tr>
<tr><td>SSC: n</td><td>43</td><td>0</td><td>42</td><td>0</td><td>34</td><td>0</td></tr>
<tr><td rowspan="5">Alaki et al., 2020 [58]</td><td rowspan="5">0= No caries
1= Caries</td><td rowspan="5">At 3, 6 and 12 m</td><td colspan="7">Total number of teeth in each group (N) = 60</td><td rowspan="5">The results showed that none of the teeth covered with ZC had developed recurrent caries. While for RCSC group, at 12 months follow up, 6.7% had presented with recurrent caries, but this finding was not significant (p < 0.135).</td></tr>
<tr><td></td><td colspan="2">3m</td><td colspan="2">6m</td><td colspan="2">12m</td></tr>
<tr><td>Score</td><td>0</td><td>1</td><td>0</td><td>1</td><td>0</td><td>1</td></tr>
<tr><td>ZC
n (%)</td><td>60 (100)</td><td>0</td><td>59 (100)</td><td>0</td><td>58 (100)</td><td>0</td></tr>
<tr><td>RCSC n (%)</td><td>60 (100)</td><td>0</td><td>60 (100)</td><td>0</td><td>56 (93.3)</td><td>4 (6.7)</td></tr>
<tr><td rowspan="5">Nischal et al., 2020 [60]</td><td rowspan="5">0= No caries
1=Recurrent caries</td><td rowspan="5">At 3, 6 and 9 m</td><td colspan="7">Total number of teeth in each group (N) = 15</td><td rowspan="5">The results showed that ZCs did not develop secondary caries. When all groups were compared, there was a statistically significant difference (p value = 0.012).</td></tr>
<tr><td></td><td colspan="2">3m</td><td colspan="2">6m</td><td colspan="2">9m</td></tr>
<tr><td>Score</td><td>0</td><td>1</td><td>0</td><td>1</td><td>0</td><td>1</td></tr>
<tr><td>RCSC
n (%)</td><td>15 (100)</td><td>0</td><td>15 (100)</td><td>0</td><td>11 (73.4)</td><td>4 (26.6)</td></tr>
<tr><td>ZC
n (%)</td><td>15 (100)</td><td>0</td><td>15 (100)</td><td>0</td><td>15 (100)</td><td>0</td></tr>
</table>

	**Luxa Crown n (%)**	15 (100)	0	15 (100)	0	15 (100)	0	

Abbreviation: EZP, EZ Pedo crown; NSZ, NuSmile Zirconia crown; SSC, Stainless steel crown; ZC, Zirconia crown; RCSC, Resin composite strip crown; DCR, Direct resin composite restoration.

Supplemental Table S9. Summary of the included studies' findings about the crown contour of zirconia crowns for primary teeth.

Study	Assessment Method	Time of Evaluation	Results				Summary of Results
Holsinger et al., 2016 [44]	0= Crown is cosmetic, natural looking.<br>1= Size/shape is acceptable, not ideal<br>2= Crown not esthetic, detracts from appearance of the mouth	(Retrospective study)<br>2010– 2014	**Total number of EZP (N) = 44**				The results showed that the majority of the EZP (89%) were cosmetic and natural-looking whereas fewer were acceptable (9%) and not esthetic (2%).
				**0**	**1**	**2**	
			**n (%)**	39 (89%)	4 (9%)	1 (2%)	
Yanover et al., 2020 [63]	0= Cosmetic<br>1= Acceptable<br>2= No aesthetic	At 6-10.4m<br>12.5-17.9m<br>18.4-33.8m	**Total number of ZC (N) = 131**				According to the crown contour, the teeth rated as cosmetic were 55% (72 teeth), while 35% (47 teeth) appeared acceptable, and 9% (12 teeth) considered as not aesthetic crowns.
				**0**	**1**	**2**	
			**n (%)**	72 (55)	47 (35)	12 (9)	

Abbreviation: EZP, EZ Pedo crown.
